# Expression and Possible Role of Silent Mating Type Information Regulation 2 Homolog 1 in Post-necrotizing Enterocolitis Stricture *in vivo* and *in vitro*

**DOI:** 10.3389/fped.2022.836128

**Published:** 2022-07-25

**Authors:** Rui Chen, Chengjie Lv, Yun Zhao, Weizhong Gu, Luyin Zhang, Bo Shi, Jingfa Tou

**Affiliations:** ^1^Department of Neonatal Surgery, Children’s Hospital Affiliated to Medical College of Zhejiang University, National Center for Clinical Medicine of Children’s Health and Disease, Hangzhou, China; ^2^Department of Pathology, Children’s Hospital Affiliated to Medical College of Zhejiang University, National Center for Clinical Medicine of Children’s Health and Disease, Hangzhou, China; ^3^Department of Pediatric Surgery, Affiliated Hangzhou First People’s Hospital, Zhejiang University School of Medicine, Hangzhou, China

**Keywords:** SIRT1 (silent mating–type information regulation 2 homolog-1), necrotizing enterocolitis (NEC), intestinal stenosis, intestinal fibrosis, TGF-β1

## Abstract

**Purpose:**

To investigate the expression and possible role of Sirtuin1 or Silent mating–type information regulation 2 homolog-1 (SIRT1) in post-necrotizing enterocolitis stricture.

**Materials and Methods:**

The expression characteristics of SIRT1 and TGF-β1 in post-necrotizing enterocolitis stricture were detected by immunohistochemistry. The siRNA-SIRT1 was used to inhibit the expression of SIRT1 in intestinal epithelial cells-6 (IEC-6), and qRT-PCR, WB, and ELISA were utilized to detect the changes of Transforming growth factor-β1 (TGF-β1), nuclear factor (NF)-κB, tumor necrosis factor-α (TNF-α), tight junction protein-1 (ZO-1), and vascular endothelial growth factor (VEGF) expressions. The IEC-6 cell proliferation and migration ability were tested *via* CCK8 kit and Transwell test. The expression of E-cadherin and Vimentin in cells was detected by immunofluorescence.

**Results:**

The CRP, IL-6, IL-10, and IFN-γ in the serum of Necrotizing enterocolitis (NEC) intestinal stenosis patients were significantly higher than the reference values. The SIRT1 protein was under-expressed and the TGF-β1 protein was overexpressed in NEC intestinal stenosis tissue. And the expression of SIRT1 was negatively correlated with TGF-β1. At the time of diagnosis of NEC, the expression of SIRT1 decreased in children with respiratory distress syndrome and CRP level increased. After inhibiting the expression of SIRT1 in IEC6 cells, the expression levels of TGF-β1, Smad3, and NF-κB were decreased, and the expression of ZO-1 was also decreased. The proliferation and migration ability of IEC6 cells was decreased significantly, and the expression of E-cadherin and Vimentin proteins in IEC6 cells did not change significantly.

**Conclusion:**

Promotion of intestinal fibrosis by inflammation may be the mechanism of post-necrotizing enterocolitis stricture. SIRT1 may be a protective protein of NEC. The probable mechanism is that SIRT1 can regulate intestinal fibrosis and can protect the intestinal mucosal barrier function to participate in the process of post-necrotizing enterocolitis stricture.

## Introduction

Necrotizing enterocolitis (NEC) is a multi-factorial illness with a common acute abdomen in neonates that mainly affects premature infants with major manifestations being intestinal inflammatory necrosis (1). The incidence of NEC is approximately 5–10% in very low–birth weight (VLBW) infants, and the incidence is highest in extremely low–birth weight (ELBW) infants (2). Owing to medical advancements in treating perinatal and premature infants, the survival of premature and low birth weight infants has improved significantly. Despite significant advancements in research and treatment of NEC, the incidence of complications on successful NEC treatment has increased owing to a boom in the number of premature and low birth weight babies. Intestinal stenosis, the most common complication of NEC, is a secondary change in intestinal tissue during the course of NEC that can occur after conservative treatment of NEC. Stenosis may occur in the distal intestinal canal after enterostomy of NEC. Proximal intestinal canal after enterostomy also can occur, but it is rarely seen in clinical practice (3). The incidence of NEC secondary intestinal stricture is 2.9–57%, mainly in the terminal ileum, ascending colon, and splenic flexure of colon, with colon stricture being the most common. The pathogenesis of NEC secondary intestinal stenosis is still uncertain due to the deposition of extracellular matrix (ECM) and abnormal expression of collagen fibers, which result in intestinal stenosis in cases of inflammatory reaction in the intestinal canal (4). Another potential mechanism is when mechanical compression of the intestinal duct by intraperitoneal adhesion triggers the ischemic injury of intestinal tissue. Our research was conducted to explain the molecular mechanism of NEC secondary intestinal stricture and to search for targeted proteins or genes that can effectively prevent or alleviate intestinal stricture.

Intestinal stenosis is one of the most evident symptoms of inflammatory bowel disease (IBD), mainly due to the repeated stimulation of chronic inflammation in intestinal tissue with intestinal fibrosis (5). Activation of inflammatory signal pathways and abnormal expression of fibrosis-related cytokines are the main molecular mechanisms of intestinal fibrosis. Transforming growth factor-β1 (TGF-β1) signal pathway activation is the most important pathway in the pathogenesis of intestinal fibrosis (6). Therefore, we speculated that TGF-β1 signaling pathway activation also appears in NEC secondary intestinal stenosis. Sirtuin1 or Silent mating–type information regulation 2 homolog-1 (SIRT1) is a histone deacetylase that curbs the release of inflammatory mediators and downsizes the activity of inflammatory factors in inflammatory responses that provide protective significance for cell damage. Studies have found that SIRT1 can curb inflammation and can downsize the occurrence and development of liver and kidney fibrosis (7, 8). In this research, the expression of SIRT1 in NEC secondary intestinal stenosis was initially obtained through experiments. The possible influence of SIRT1 on NEC secondary intestinal stenosis was explained by cytological experiments. Therefore, we put forward a hypothesis of low expression of SIRT1 protein and overexpression of TGF-β1 protein in NEC secondary intestinal stricture tissue, where SIRT1 and TGF-β1 expressions are negatively correlated. The low expression of SIRT1 may promote the occurrence of intestinal stricture secondary to NEC. SIRT1 has a protective effect on intestinal epithelial cells and can regulate the expression of inflammatory signaling pathways and related proteins to participate in the occurrence of intestinal stricture secondary to NEC. SIRT1 may be a target for relieving intestinal stricture secondary to NEC.

## Materials and Methods

### Chemicals and Materials

Mice anti-human SIRT1 antibody (Abcam, ab110304, United States), rabbit Anti-Human Restructuring Anti-Smad3 (USA abcam, ab40854), rabbit anti-human nuclear factor p65(NF-κB p65) antibody C-20 (United States Santa Cruz Biotechnology, sc-372), rabbit anti-human TGF-β1 antibody V (United States Santa Cruz Biotechnology, sc-146), mouse monoclonal antibody tumor necrosis factor-α (TNF-α) (USA abcam, ab1793), mouse monoclonal antibody TNF-α (USA Abcam, ab1793), and rabbit anti-human ZO-1 antibody (United States Santa Cruz Biotechnology, sc-33725) were used. The CCK8 kit (Tongren Chemical, Japan), the vascular endothelial growth factor (VEGF)-ELISA kit (Elabscience, China), the gene sequence of siRNA-SIRT1 (Invitrogen Company, USA), the epithelial–mesenchymal transition (EMT) detection marker E-cadherin (USA abcam, ab40772), and Vimentin (USA abcam, ab92547) were used. All other reagents were used at an analytical grade.

### Clinical Specimens

Inclusion criteria: No prior history of abdominal surgery or abdominal puncture. Patients with a definite diagnosis of NEC before surgery had intestinal stricture confirmed by gross observation during surgery, and intestinal stricture was confirmed by pathological specimens after surgery. Patients have complete clinical data including gender, gestational age, birth weight, preoperative blood routine, preoperative blood gas, and other information.

Diagnostic criteria of NEC: neonates, especially preterm infants, have clinical symptoms of feeding intolerance, vomiting, abdominal distension, hematochezia, and systemic infection. Radiographs of the abdomen showed dilatation and stiffness of the bowel and (or) pneumatosis of the intestinal wall and portal vein. Feeding intolerance, abdominal distention, and bloody stools were observed after 8–10 days of age. The pathognomonic findings on abdominal radiography were pneumatosis intestinalis, portal venous gas, or both (1).

Diagnostic criteria of NRDS: neonatal respiratory distress syndrome (NRDS), also known as hyaline membrane disease, is one of the most common respiratory diseases in neonates and is mainly caused by immature neonatal lung development and lack of pulmonary surfactant (PS). Clinical symptoms are characterized by respiratory distress and progressive aggravation that appear shortly after birth, usually with bruising and moaning as the first symptoms in clinical practice (9).

Exclusion criteria: patients with an unclear history of NEC, idiopathic perforation, sepsis not caused by NEC, and patients with intraoperative intestinal stenosis not identified as congenital Hirschsprung’s disease or suspected congenital Hirschsprung’s disease.

Anemia: Neonates with venous blood Hb ≤ 130 g/L within 1 week after birth, capillary blood Hb ≤ 145 g/L after birth < 100 g/L, can be diagnosed as anemia.

Sepsis: 1. Neonatal EOS: (1) Suspected diagnosis of any of the following within 3 days of age, ➀ abnormal clinical manifestations, ➁ mother with chorioamnionitis, ➂ premature birth with PROM ≥ 18 h. If there are no abnormal clinical manifestations, blood cultures are negative, and two consecutive non-specific blood tests with an interval of 24 h are < 2 positive, sepsis can be excluded. (2) The clinical diagnosis is clinically abnormal, and any one of the following conditions is met at the same time: ➀ non-specific blood test > 2 positive, ➁ cerebrospinal fluid test shows purulent meningitis changes, and ➂ pathogenic bacterial DNA is detected in the blood. (3) The diagnosis is confirmed as having clinical manifestations, and the culture of blood or cerebrospinal fluid (or other sterile cavity fluid) is positive. 2. Neonatal 1. OS: The clinical diagnosis and confirmed diagnosis are both > 3 days old, and other conditions are the same as those of neonatal EOS.

Neonatal asphyxia: Newborns should still be assessed for Apgar scores after birth, and umbilical artery blood gas analysis should be done immediately after birth in hospitals with secondary or higher levels or conditions. Apgar scores should be combined with blood gas results to make a diagnosis of asphyxia. (1) Mild asphyxia: Apgar score 1 min ≤ 7 points, or 5 min < 7 points, with umbilical artery blood pH < 7.2. (2) Severe asphyxia: Apgar score 1 min ≤ 3 points or 5 min < 5 points, with umbilical artery blood pH < 7.0.

According to inclusion and exclusion criteria, 43 patients with post-necrotizing enterocolitis stricture who were treated by neonatal surgery in our hospital from January 2019 to December 2020 were enrolled in our study. We selected the narrow bowel tissue of these children as the study objects and the marginal bowel tissue as the control. All experiments performed in the present study had been approved by the Ethics Committee of The Children’s Hospital Zhejiang University School of Medicine, and the ethical review number is 2021-IRB-048.

### Immunohistochemistry Analysis (EnVision Two-Step Method)

Xylene dewaxing was done two times for 10 min each; anhydrous ethanol was added to the slices for 3 min two times; 95% ethanol was then added for 2 min two times; 80% ethanol was added for 2 min; 70% ethanol was added for 2 min; and for natural hydration, the sample was distilled and washed. Antigen repair: was performed at a high temperature and high pressure. The antigen repair solution (0.01 M Ethylene Diamine Tetraacetic Acid (EDTA) buffer solution, pH 9.0) was added to the slices for 2 min and 30 s after drying. These were kept warm for 4 min after the power was turned off, followed by flushing with water to room temperature. Then, they were washed in distilled water and a circle was made. A 3% H_2_O_2_ aqueous solution was used to block the endogenous peroxidase for 10 min. Three washes with phosphate-buffered solution (PBS) for 5 min followed. A proper amount of diluted primary antibody (the dilution ratio of SIRT1 and TGF-β1 was 1:150) was added, followed by incubation at 37°C for 60 min. The slices were rinsed with PBS for 5 min 3 times. The antibody against rabbit IgG-HRP polymer was incubated at 37°C for 30 min, then washed with PBS 3 times for 5 min each. The slices were incubated with a 3,3’-diaminobenzidine(DAB) chromogenic solution (pre-use configuration) for 1 min, and the reaction was manipulated under a microscope. Tap water flushing was used to terminate the reaction. The nuclei were restained with a Harris hematoxylin solution for 3 min (differentiation was confirmed), dehydrated with 95 and 100% ethanol, and transparent xylene, and sealed with neutral gum. Immunohistochemical positive results were yellow or yellowish-brown with a blue nuclear lining. The SIRT1 and TGF-β1 positive staining area was quantified using the average optical density (AOD) function in the ImageJ Analyzer software. AOD = IOD (Integrated Optical Density) SUM/Positive tissue area.

### Cell Culture and Cell Transfection

The cell lines small intestinal epithelial cells (IEC-6) of SD female rats were purchased from ATCC cell bank. The IEC-6 cells were cultured according to the supplier’s recommendations. The IEC-6 cells were cultured in Dulbecco’s modified eagle medium (DMEM) supplemented with 10% fetal bovine serum, 1% penicillin/streptomycin, and 0.1 U/ml bovine insulin as described. SIRT1 was knocked down by siRNA in IEC-6 cells according to the manufacturer’s instructions. IEC-6 cells were seeded in 6-well plates, and then a specific SIRT1 siRNA and control negative (NC) siRNA were transfected with Lipofectamine RNAiMAX. After 48 h, total RNA of cells was extracted for qPCR detection.

### Quantitative Real-Time PCR Analysis

Intestinal epithelial cells-6 (IEC-6) were inoculated into a 6-well cell culture plate at a density of 50,000 cells/well. RNA transfection occurred after 18 h for the NC group and the experimental group (siRNA-SIRT1). Two replicates were performed for each group. The dosages of Lipofectamine RNAiMAX and RNA used were 7.5 μl/well and 75 pmol/well, respectively. After 48 h of transfection, total RNA of cells was extracted for qRT-PCR detection (10). We constructed the siRNA gene sequence targeting SIRT1, and the gene sequence of SIRT1 is sense 5’-CAGGAAUCCAAAGGAUAAUTT-3’, and antisense 5’-AUUAUCCUUUGGAUUCCUGTT-3’. The qRT-PCR primer sequences are shown in Table 1. Relative mRNA levels were calculated based on the CT values, corrected for GAPDH expression, according to the equation: 2^–Δ*CT*^ (ΔCT = CT gene of interest – CT of GAPDH) (11).

**TABLE 1:** The qRT-PCR gene sequences.

Gene name	Primer sequence
Rat-ZO-1-F1	TTCGCCTGAAACAAACCCAG
Rat-ZO-1-R1	CTTGTGATACGTGCGAGGTG
Rat-VEGF-F1	GGAACTAGACCTCTCACCGG
Rat-VEGF-R1	CTCTCCCTTCATGTCAGGCT
Rat-TGF-β1-F1	GACCGCAACAACGCAATCTA
Rat-TGF-β1-R1	ACTGCTTCCCGAATGTCTGA
Rat-TNF-α-F1	TCCCAGAAAAGCAAGCAACC
Rat-TNF-α-R1	TAGACAGAAGAGCGTGGTGG
Rat-NF-κB-F1	CGTGAGGCTGTTTGGTTTGA
Rat-NF-κB-R1	TCTGCCCTCCTGACTCTACT
Rat-Smad3-F1	CATGGGCAAATGAAAGGGCT
Rat-Smad3-R1	CCAGGGTGAAGATGACAGGT
Rat-GAPDH-F1	TGCTGAGTATGTCGTGGAGTCT
Rat-GAPDH-R1	CAGTCTTCTGAGTGGCAGTGAT
Rat-SIRT1-F1	TACCCCATGAAGTGCCTCAA
Rat-SIRT1-R1	CATCGCAGTCTCCAAGAAGC

### Western Blotting Analysis

Intestinal epithelial cells-6 (IEC-6) were inoculated into a 6-well cell culture plate at a density of 50,000 cells/well. RNA transfection occurred after 18 h for the NC group and the experimental group (siRNA-SIRT1). Two replicates were performed for each group. The dosages of Lipofectamine RNAiMAX and RNA used were 7.5 μl/well and 75 pmol/well, respectively. After 48 h of transfection, proteins were obtained from those IEC-6 cells. To extract the total protein, the cell lysates were prepared by centrifuging at 12,000 rpm and 4°C for 10 min, and the supernatant was obtained. The total protein concentration was measured using the Bradford method, and the OD value was measured at 595 nm using an enzyme marker. The standard curve and the total protein concentration of the samples were calculated. According to the calculated total protein concentration, the total protein concentration of each group was adjusted with 1 × PBS; and 4 × SDS loading buffer was added and the mixture was heated for 10 min at 100°C. Then, it was centrifuged at 12,000 × g for 1 min and supernatant samples were collected. Samples were separated using 10% SDS-PAGE. The Tianneng VE-180 vertical electrophoresis tank was used to run the gel for 30 min at 80 V and 70 min at 120 V. The membranes were blocked with non-fat milk powder for 1–1.5 h at room temperature and then were incubated at 4°C overnight with the corresponding primary antibodies(SIRT1 1:1000 dilution, TGF-β1 1:1000 dilution, Smad3 1:1000 dilution, NF-κB p65 1:2000 dilution, TNF-α 1:1000 dilution, ZO-1 1:200 dilution). After being rinsed with TBST four times for 5 min each, the membranes were incubated with the secondary antibodies for 1 h at 37°C and then washed with TBST again, as previously described. Three replicates per sample were performed to exclude accidental results. The protein bands were visualized using an ECL chemiluminescence solution. The intensity of the immune-complexes was captured and analyzed using Image J software.

### Vascular Endothelial Growth Factor Test

Intestinal epithelial cells-6 (IEC-6) were inoculated into two 24-well cell culture plates. RNA transfection occurred after 18 h for the NC group and the experimental group (siRNA-SIRT1). The experiment was done in duplicate per group. The dosages of Lipofectamine RNAiMAX and RNA used were 1.5 μl/well and 15 pmol/well, respectively. After 48 h of transfection, intracellular vascular endothelial growth factor (VEGF) and extracellular VEGF were determined using enzyme-linked immunosorbent assay kits according to the manufacturer’s instructions.

### Cell Viability Assay

Intestinal epithelial cells-6 (IEC-6) were inoculated into 96-well plates at a density of 3,000 cells/well. RNA transfection occurred after 18 h for the NC group and the experimental group (siRNA-SIRT1). Each experiment was repeated three times per group. The dosages of Lipofectamine RNAiMAX and RNA used were 0.3 μl/well and 3 pmol/well, respectively. After 72 h of transfection, the medium of each well was replaced with DMEM complete medium containing 10% CCK8, and the plates were further incubated for 1 h. The absorption values at 450 nm were detected using an enzyme-labeling instrument.

### Cell Proliferation Assay

Intestinal epithelial cells-6 (IEC-6) were inoculated into 24-well cell culture plates (1 × 10^5^ cells/well). Cell transfection was the same as cell viability assay. The dosages of Lipofectamine RNAiMAX and RNA used were 1.5 μl/well and 15 pmol/well, respectively. After 48 h of transfection, 20,000 cells were collected and cultured in the upper chamber of a Transwell in 100 μl of medium with no serum. Three Transwell plates were used for each group. Medium containing 10% of serum (600 μl) was added to the lower compartment. After 24 h, the chamber was fixed with 4% paraformaldehyde, then the cells were gently removed from the upper membrane, and the cells in the lower membrane were stained with 4’,6-diamidino-2-phenylindole(DAPI). The cells of the stained lower membrane were photographed using a fluorescence microscope. Five fields of view were randomly selected for each compartment, using a 20 × objective lens. The cell number in each field of view was counted using cell counting software, and the average number counted in five fields per well was used for data analysis.

### Epithelial–Mesenchymal Transition (EMT) Detection

IEC-6 cells were inoculated into 24-well cell culture plates at 3,000 cells/well. Cell transfection was the same as cell viability assay. Two experiments were done per group. The dosages of Lipofectamine RNAiMAX and RNA used were 0.75 μl/well and 7.5 pmol/well, respectively. After 48 h of transfection, the medium was discarded, the cells were washed once with PBS, and fixed with 4% paraformaldehyde for 30 min. The fixating solution was discarded, and a 0.5% Triton solution was added for 15 min. The Triton solution was discarded, and the cells were washed 3 times with PBS. The sealing solution 1% albumin from bovine serum (BSA) was added to the plate (100 μl/well), followed by sealing at room temperature for 1 h. The sealing liquid was discarded, and cells were washed 3 times with PBS. E-Cadherin and vimentin primary antibodies were diluted (1:200) and added to the plate of the corresponding group (100 μl/well), which was incubated at 4°C overnight. WB Antibody Diluent was discarded, followed by 5 washes with PBS. The FITC-labeled secondary antibody was diluted (1:200) and added to the plate (100 μl/well), and incubated at room temperature for 1 h. The secondary antibody Dilution Buffer was discarded, followed by 5 washes with PBS. DAPI solution was added to stain cells for 3 min, and pictures were taken using a fluorescence microscope.

### Statistical Analysis

GraphPad Prism 7.0 and SPSS 23.0 were used for data analysis. All values are presented as the mean ± standard deviation (SD). Student’s *t*-test was used to compare means between groups. Data not conforming to a normal distribution were represented by M (Q1, Q3), and a Mann–Whitney *U* test was adopted. *p* < 0.05 indicated statistically significant differences.

## Results

### Clinical Features of Children With Necrotizing Enterocolitis (NEC) Secondary Intestinal Stricture

Among the 43 children with NEC secondary intestinal stricture, 21 were boys and 22 were girls. There were 39 premature infants, 4 full-term infants, 28 cesarean sections, and 15 natural births. There were 36 low birth weight infants. The mean birth weight and gestational age of 43 infants with NEC secondary enterostricture were 1771 ± 92.31 g and 32.28 ± 0.48 w. The body weight at diagnosis of NEC was 2125 ± 69.20 g, and the interval between diagnosis of intestinal stenosis and diagnosis of NEC was 21.79 ± 1.89 days. The mean age of the first admission to our hospital was 29.67 ± 3.336 days, and the mean weight was 2,240 ± 69.86 g. The mean age and weight of children with NEC secondary intestinal stenosis at the time of operation were 46.4 ± 3.093 days and 2,590 ± 67.94 g, respectively.

Among the children with intestinal stenosis, 26 cases were premature infants who underwent cesarean section. In total, 16 cases of premature birth due to premature rupture of membranes, 4 cases of premature birth due to fetal intrauterine distress and fetal growth restriction, 5 cases of premature birth due to twin pregnancy, 3 cases of premature birth due to mother’s cholestasis, and 3 cases of premature birth due to placenta previa and placental separation. There are also other causes such as mother’s heart disease, pregnancy-induced hypertension, and severe eclampsia. During the analysis of the main causes of premature birth in children, we found that the main causes of preterm birth in children with intestinal stricture are premature rupture of membranes and intrauterine distress, twin pregnancy, maternal cholestasis during pregnancy, and placenta previa.

Postnatal Apgar score (1 min) was less than 4 in 2 cases, 5 cases with 5–6 points, 27 cases with 7–10 points, and 9 cases with unclear score. Postnatal Apgar score (5 min) was less than 4 in 0 cases, 1 case with 5–6 points, 33 cases with 7–10 points, and 9 cases with an unclear score. In total, 23 children were transferred to the NICU ward for treatment after birth. There were 10 infants diagnosed with neonatal asphyxia after birth, and 33 infants with no diagnosis of neonatal asphyxia. There were 15 infants diagnosed with neonatal respiratory distress syndrome after birth and 28 infants without respiratory distress syndrome. There were 21 infants with neonatal sepsis and 22 infants without a diagnosis of sepsis. There were 18 infants who required mechanical ventilation after birth, and 25 infants who were not mechanically ventilated. There were 11 children with congenital heart disease at the same time, and 32 children were not diagnosed with congenital heart disease. There were 22 children diagnosed with anemia during NEC, and 21 without anemia. There were 16 children who received a blood transfusion before surgery and 27 children who did not receive a blood transfusion.

When the children were diagnosed with NEC, 40 children had serum CRP ≥ 8.0 mg/ml, and 3 children had serum CRP < 8.0 mg/ml. During the duration of NEC, the mean value of serum CRP was 53.22 ± 6.56 mg/L, and the duration of continuous elevation of CRP was 11.74 ± 1.18 days. Among 43 patients, 16 children underwent flow cytometry during the period of diagnosis of NEC. The results of these cytokines were summarized and showed that IL-2, IL-4, and TNF did not increase and that IL-6, IL-10, and IFN-γ increased significantly. The specific values were IL-2 2.7 (2.4, 3.725) pg⋅ml^–1^, IL-4 2.5 (1.875, 2.775) pg⋅ml^–1^, IL-6 44.7 (19.5, 1485) pg⋅ml^–1^, IL-10 13 (6.725, 59.33) pg⋅ml^–1^, TNF 1.8 (1.425, 2.75) pg⋅ml^–1^, IFN-γ 23.75 (3.75, 49.78) pg⋅ml^–1^. The reference ranges for these cytokines are IL-2 (1.1–9.8 pg⋅ml^–1^), IL-4 (0.1–3.0 pg⋅ml^–1^), IL-6 (1.7–16.6 pg⋅ml^–1^), IL-10 (2.6–4.9 pg⋅ml^–1^), TNF (0.1–5.2 pg⋅ml^–1^), IFN-γ (1.6–17.3 pg⋅ml^–1^). Bell staging included 18 children with stage I, 23 with stage II, and 2 with stage III.

By summarizing the clinical characteristics of children with NEC secondary intestinal stricture, we found that the children with intestinal stricture were mainly premature infants and low birth weight infants. Hypoxia and inflammation may be the main risk factors for NEC intestinal stenosis in premature infants.

In this study, a total of 80 NEC secondary intestinal stricture patients treated by neonatal surgery in our hospital from June 2018 to October 2020 were counted, and only 3 patients underwent enterostomy, and there were no cases of death or abandonment of treatment. During the same period, 112 children underwent emergency necrotic bowel resection and enterostomy because of NEC, of which 10 patients died and gave up treatment. The time interval from diagnosis of NEC to emergency enterostomy is usually 1-2 days, the shortest is half a day, and the longest is 12 days. The average time interval of NEC enterostomy return was 96.21 days. The overall treatment effect of children with NEC secondary intestinal stricture was significantly better than that of children with NEC intestinal necrosis requiring enterostomy.

### Pathological Features of Intestinal Stricture Secondary to Necrotizing Enterocolitis (NEC)

**FIGURE 1:**
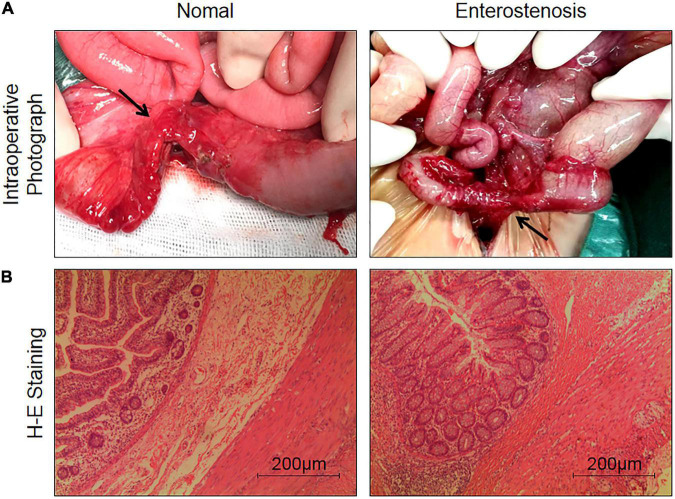
The characteristics of pathological changes in post-necrotizing enterocolitis stricture. **(A)** Intraoperative photograph of post-necrotizing enterocolitis stricture tissue (the black arrow indicates the intestinal stenosis). **(B)** Comparison of H and E staining pictures of intestinal normal tissue and post-necrotizing enterocolitis stricture tissue (5 × 10 multiplying).

Intraoperative photographs of infants with NEC secondary intestinal stricture showed inflammatory changes in the intestinal tract, marked fibrous hyperplasia, and small intestinal lumen obstruction (Figure 1A). Hand E staining images of narrow intestinal tissue showed atrophy of mucosal layer, small intestinal lumen, obvious hyperplasia of submucosal fibers, and obvious infiltration of inflammatory cells (Figure 1B). Combined with the results of previous CRP elevation in these infants, we could preliminarily conclude that inflammatory response and fibrosis were the possible mechanisms of intestinal stenosis secondary to NEC.

### Expression Characteristics of Sirtuin1 or Silent Mating–Type Information Regulation 2 Homolog-1 (SIRT1) Protein in Intestinal Stricture Secondary to Necrotizing Enterocolitis (NEC)

**FIGURE 2:**
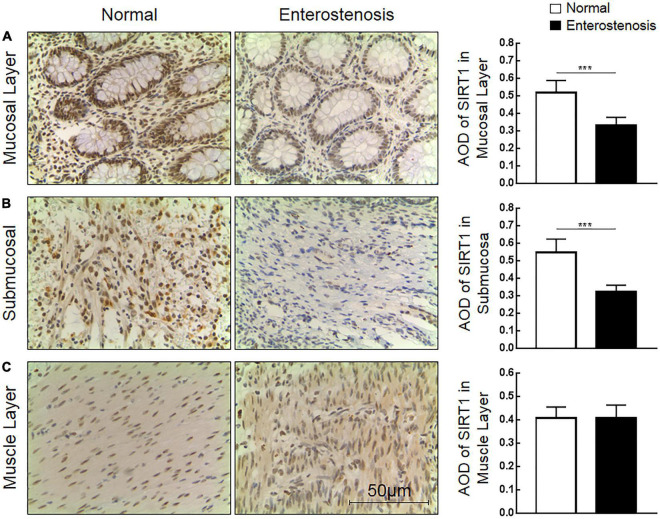
The expression characteristics of Sirtuin1 or Silent mating–type information regulation 2 homolog-1 (SIRT1) protein in post-necrotizing enterocolitis stricture tissue. **(A)** Expression of SIRT1 protein in the intestinal mucosal layer. **(B)** Expression of SIRT1 protein in the submucosal layer of the intestine. **(C)** Expression of SIRT1 protein in the muscularis of the intestine. (***Denotes the enterostenosis groups compared with the normal groups, *n* = 43, *p* < 0.001).

The expression location of SIRT1 protein is mainly in the nucleus, with little expression in the cytoplasm, showing brownish red granules. Expressions of SIRT1 protein occur in mucosal layer (Figure 2A), submucosal layer (Figure 2B), and muscular layer (Figure 2C) of the intestinal wall tissue. Expression in the narrow section of the intestine was lower than that in the control group (Table 2). The SIRT1 protein expression in the mucosa and submucosa of the narrow intestine was lower than that in the control group, and the SIRT1 protein expression in the muscle layer was not significantly different between the two groups.

**TABLE 2:** The expression of Sirtuin1 or Silent mating–type information regulation 2 homolog-1 (SIRT1) and transforming growth factor-β1 (TGF-β1) proteins in the two groups of intestinal tissues.

Protein	Anatomical structure	Normal intestinal tissue (*n* = 43)	Narrow intestinal tissue (*n* = 43)	*P*-value
SIRT1	Mucous layer	0.52 (0.47,0.58)	0.32 (0.29,0.36)	< 0.0001
	Submucosa	0.55 (0.47,0.59)	0.32 (0.29,0.34)	< 0.0001
	Muscular layer	0.42 (0.37,0.44)	0.41 (0.37,0.44)	0.7319
TGF-β1	Mucous layer	0.27 (0.21,0.29)	0.56 (0.52,0.65)	< 0.0001
	Submucosa	0.27 (0.26,0.31)	0.46 (0.43,0.52)	< 0.0001
	Muscular layer	0.21 (0.19,0.22)	0.31 (0.28,0.32)	< 0.0001

### Expression Characteristics of Transforming Growth Factor-β1 (TGF-β1) Protein in Necrotizing Enterocolitis (NEC) Secondary Intestinal Stricture

**FIGURE 3:**
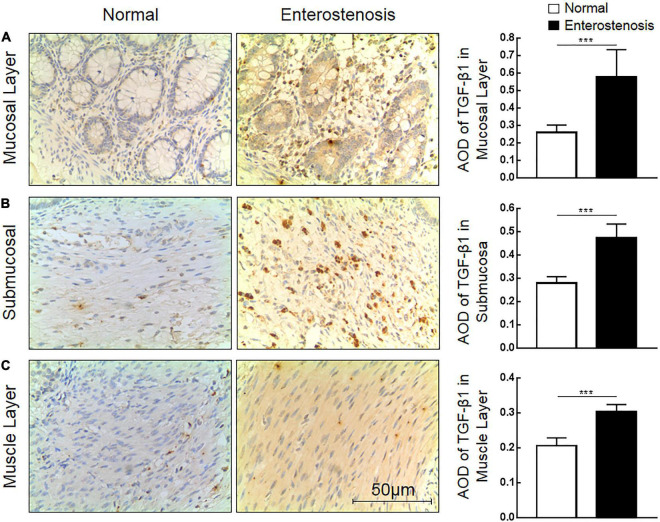
The expression characteristics of transforming growth factor-β1 (TGF-β1) protein in post-necrotizing enterocolitis stricture tissue. **(A)** Expression of TGF-β1 protein in the intestinal mucosal layer. **(B)** Expression of TGF-β1 protein in the submucosa of the intestine. **(C)** Expression of TGF-β1 protein in the muscularis of the intestine. (***Denotes the enterostenosis groups compared with the normal groups, *n* = 43, *p* < 0.001).

The expression location of TGF-β1 protein is mainly in the cytoplasm with brownish-yellow granules. Expressions of TGF-β1 protein occur in the mucosal layer (Figure 3A), submucosal layer (Figure 3B), and muscular layer (Figure 3C) of intestinal wall tissue. In the narrow section of intestinal tissue, expression was lower than that in the control group (Table 2). The expression of TGF-β1 protein in the mucosa, submucosa, and muscularis of the narrow intestine was increased compared with that in the control group.

### Clinical Significance of Sirtuin1 or Silent Mating–Type Information Regulation 2 Homolog-1 (SIRT1) Protein and Transforming Growth Factor-β1 (TGF-β1) Protein Expression in Necrotizing Enterocolitis (NEC) Secondary Intestinal Stricture

According to the expression characteristics of SIRT1 protein and TGF-β1 protein in the mucosal layer, we selected the clinical characteristics of SIRT1 protein expression in the mucosal layer of intestinal tissue of 43 infants with NEC secondary intestinal stricture. There was no significant difference in the expression of SIRT1 protein in gender, delivery mode, premature birth, and low birth weight. The expression of SIRT1 protein was not affected by anemia, blood transfusion, sepsis, parity, NICU, mechanical ventilation, CHD, Apgar score (1 min), and neonatal asphyxia in 43 children diagnosed with NEC (Table 3). The expression of SIRT1 protein in NEC patients with NEONATAL distress syndrome was lower than that in infants without neonatal distress syndrome. The expression of SIRT1 protein was lower in infants with elevated CRP. BELL staging had no significant effect on SIRT1 protein expression (Table 3). The expression level of TGF-β1 protein in infants with BELL stage II was significantly higher than that in infants with BELL stage I (Table 3). Other factors had no significant effect on TGF-β1 protein expression (Table 3).

**TABLE 3:** The relationship between Sirtuin1 or Silent mating–type information regulation 2 homolog-1 (SIRT1) and transforming growth factor-β1 (TGF-β1) protein expression and clinical features in mucous layer of post-necrotizing enterocolitis stricture tissues.

Clinical features		SIRT1			TGF-β 1	
		Cases (*n*)	AOD ($\bar{x}$ ± s)	*P*-value	AOD ($\bar{x}$ ± s)	*P*-value
Gender	Male	21	0.3186 ± 0.01015	0.1359	0.5833 ± 0.03964	0.8132
	Female	22	0.3405 ± 0.01018		0.5718 ± 0.02839	
Delivery mode	Cesarean	28	0.3304 ± 0.009048	0.9137	0.5546 ± 0.02526	0.1965
	Eutocia	15	0.3287 ± 0.01279		0.62 ± 0.04927	
Premature	Yes	39	0.3315 ± 0.007909	0.4555	0.5862 ± 0.02576	0.2605
	No	4	0.3125 ± 0.01377		0.4925 ± 0.03924	
LBW	Yes	36	0.3328 ± 0.008429	0.3560	0.5833 ± 0.027	0.5827
	No	7	0.3143 ± 0.01066		0.5471 ± 0.05065	
Anemia	Yes	22	0.3305 ± 0.008985	0.9246	0.5636 ± 0.03376	0.5611
	No	21	0.329 ± 0.01185		0.5919 ± 0.03446	
Transfusion	Yes	16	0.3331 ± 0.01094	0.7279	0.5688 ± 0.0381	0.7835
	No	27	0.3278 ± 0.009789		0.5826 ± 0.0312	
Sepsis	Yes	21	0.3305 ± 0.009697	0.9258	0.5695 ± 0.02995	0.7507
	No	22	0.3291 ± 0.01109		0.585 ± 0.03764	
Neonatal asphyxia	Yes	10	0.345 ± 0.01558	0.2557	0.646 ± 0.05042	0.1158
	No	33	0.3252 ± 0.008224		0.5567 ± 0.02654	
Neonatal respiratory distress syndrome	Yes	15	0.3186 ± 0.008978	0.0345	0.6227 ± 0.04113	0.1693
	No	28	0.3507 ± 0.01097		0.5532 ± 0.0289	
Bell stage	I	18	0.3433 ± 0.01215	0.1908	0.5067 ± 0.03445	0.0274
	II	23	0.3226 ± 0.009266		0.6357 ± 0.03141	
	III	2	0.290 ± 0.020		0.545 ± 0.025	
CRP	≥8.0	40	0.3283 ± 0.007493	0.0245	0.5033 ± 0.05333	0.3668
	<8.0	3	0.3933 ± 0.01453		0.4698 ± 0.009402	
Parity	P1	19	0.3321 ± 0.009099	0.8441	0.5347 ± 0.0412	0.1746
	P2	16	0.3375 ± 0.01569		0.5888 ± 0.03022	
	P3	8	0.325 ± 0.01488		0.6563 ± 0.05137	
NICU	Yes	23	0.3404 ± 0.009648	0.2774	0.607 ± 0.0364	0.1892
	No	20	0.324 ± 0.01155		0.5435 ± 0.02904	
Mechanical Ventilation	Yes	18	0.3411 ± 0.01163	0.3505	0.6094 ± 0.04354	0.2613
	No	25	0.326 ± 0.009759		0.5544 ± 0.02652	
CHD(congenital heart disease)	Yes	11	0.3373 ± 0.0183	0.7294	0.5627 ± 0.05375	0.7231
	No	32	0.3313 ± 0.008004		0.5825 ± 0.02681	
Apgar score (1 min)	0∼6	7	0.3214 ± 0.0161	0.4965	0.6129 ± 0.0571	0.4273
	7∼10	27	0.3367 ± 0.01043		0.5637 ± 0.02742	
	Unknown	9	0.33 ± 0.01291		0.5911 ± 0.07007	
Apgar score (5 min)	0∼6	1	0.28	–	0.42	–
	7∼10	33	0.3352 ± 0.009005		0.5785 ± 0.02488	
	Unknown	9	0.33 ± 0.01291		0.5911 ± 0.07007	

Combined with the clinical trial results, we could preliminarily conclude that intestinal fibrosis is the final pathological change of NEC secondary intestinal stenosis. TGF-β1 protein, an important cytokine related to fibrosis, is highly expressed in NEC secondary intestinal stenosis. Inflammation may be involved in the occurrence of NEC secondary intestinal stenosis. The expression of SIRT1 protein, which inhibits inflammatory response, was low in NEC secondary intestinal stricture and was negatively correlated with CRP expression. These results suggested that SIRT1/TGF-β1 signal pathway may be involved in the occurrence of NEC secondary intestinal stenosis.

### Regulation of Sirtuin1 or Silent Mating–Type Information Regulation 2 Homolog-1 (SIRT1) Protein on Fibrosis-Related Proteins in Intestinal Epithelial Cells IEC6

**FIGURE 4:**
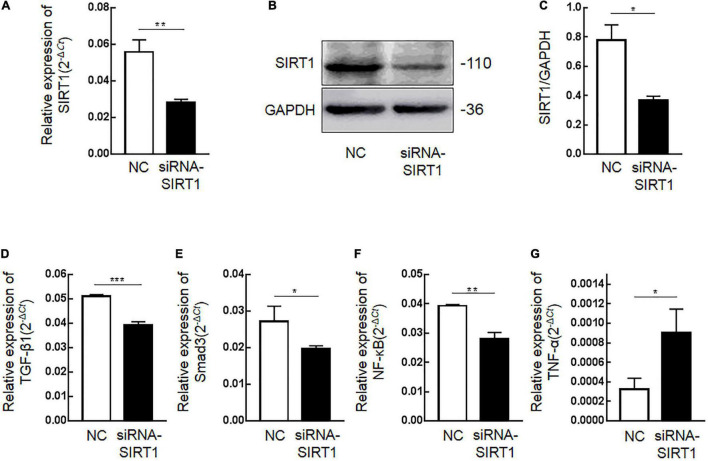
The effect of Sirtuin1 or Silent mating–type information regulation 2 homolog-1 (SIRT1) on the mRNA expression of fibrosis-related factors in intestinal epithelial cells 6 (IEC6). **(A)** After transfection of siRNA-SIRT1, the mRNA expression of SIRT1 decreased. **(B,C)** After transfection of siRNA-SIRT1, the protein expression of SIRT1 decreased. **(D)** After inhibiting the expression of SIRT1 in IEC6 cells, the mRNA expression of transforming growth factor-β1 (TGF-β1) decreased. **(E)** After inhibiting the expression of SIRT1 in IEC6 cells, the mRNA expression of Smad3 decreased. **(F)** After inhibiting the expression of SIRT1 in IEC6 cells, the mRNA expression of NF-κB p65 decreased. **(G)** After inhibiting the expression of SIRT1 in IEC6 cells, the expression of tumor necrosis factor-α (TNF-α) mRNA in the cells increased. (*Denotes the siRNA-SIRT1 groups compared with the NC groups *p* < 0.05, ** denotes *p* < 0.01, *** denotes *p* < 0.001, *n* = 3).

**FIGURE 5:**
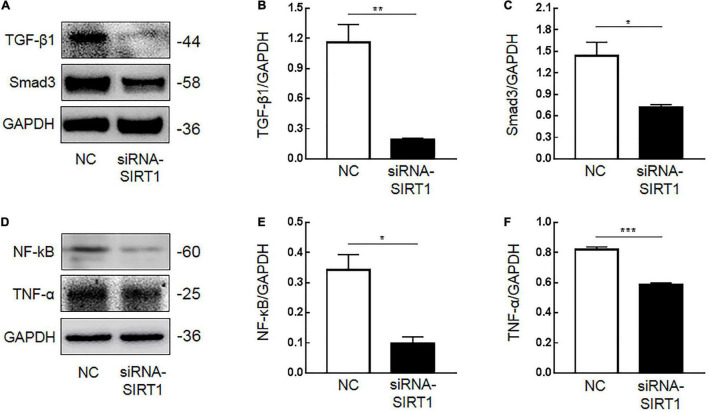
The effect of Sirtuin1 or Silent mating–type information regulation 2 homolog-1 (SIRT1) on the protein expression of fibrosis-related factors in intestinal epithelial cells 6 (IEC6). **(A,B)** After inhibiting the expression of SIRT1 in IEC6 cells, the expression of transforming growth factor-β1 (TGF-β1) protein decreased. **(A,C)** After inhibiting the expression of SIRT1 in IEC6 cells, the expression of Smad3 protein decreased. **(D,E)** After inhibiting the expression of SIRT1 in IEC6 cells, the expression of NF-κB p65 protein decreased. **(D,F)** After inhibiting the expression of SIRT1 in IEC6 cells, the expression of tumor necrosis factor-α (TNF-α) protein in the cells decreased. (*Denotes the siRNA-SIRT1 groups compared with the control negative (NC) groups *p* < 0.05, ** denotes *p* < 0.01, ***denotes *p* < 0.001, *n* = 3).

Immunohistochemical results showed that SIRT1 protein expression was decreased in mucosal epithelial cells and that TGF-β1 protein expression was increased. Therefore, we speculated that SIRT1 protein in the INTESTINAL epithelial cells IEC6 could regulate the expression of fibrotic-related proteins. The expressions of SIRT1mRNA (Figure 4A) and protein (Figures 4B,C) in IEC6 were successfully inhibited by the siRNA method. The mRNA (Figures 4D–G) and protein (Figures 5A–E) expressions of TGF-β1, Smad3, and NF-κB decreased. TNF-α mRNA (Figure 4G) expression increased, and protein (Figure 5F) expression decreased. These results confirmed that SIRT1 can regulate the expression of fibrosis-related factors TGF-β1, Smad3, NF-κB, and TNF-α in intestinal epithelial cells.

### Effects of Sirtuin1 or Silent Mating–Type Information Regulation 2 Homolog-1 (SIRT1) on Intestinal Mucosal Barrier Function

**FIGURE 6:**
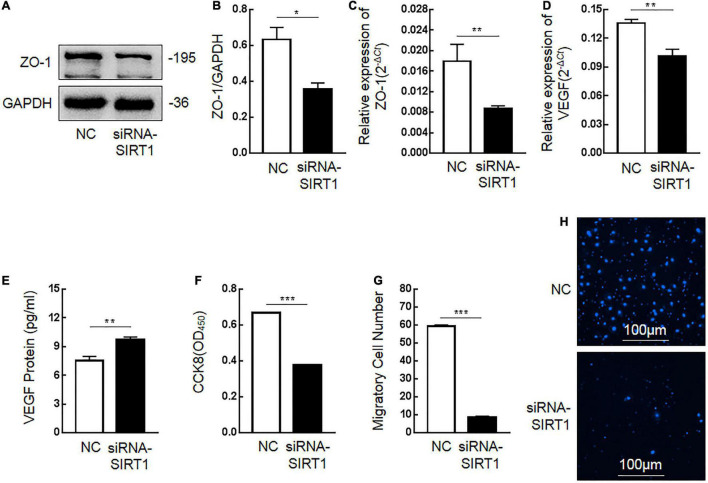
The influence of Sirtuin1 or Silent mating–type information regulation 2 homolog-1 (SIRT1) on related indexes of intestinal mucosal barrier function. **(A,B)** After inhibiting the expression of SIRT1 in intestinal epithelial cells 6 (IEC6), the expression of tight junction protein-1 (ZO-1) protein decreased. **(C)** After inhibiting the expression of SIRT1 in IEC6 cells, the expression of ZO-1 mRNA decreased. **(D)** After inhibiting the expression of SIRT1 in IEC6 cells, the expression of vascular endothelial growth factor (VEGF) mRNA decreased. **(E)** After inhibiting the expression of SIRT1 in IEC6 cells, the expression of VEGF protein in the cells increased. **(F)** After inhibiting the expression of SIRT1 in IEC6 cells, the cell proliferation ability was significantly reduced. **(G,H)** After inhibiting the expression of SIRT1 in IEC6 cells, the cell migration ability was significantly reduced. (*Denotes the siRNA-SIRT1 groups compared with the control negative (NC) groups *p* < 0.05, ** denotes *p* < 0.01, *** denotes *p* < 0.001, *n* = 3).

Tight junction protein-1 (ZO-1) and VEGF are proteins that maintain the stability of intestinal mucosal barrier function. After SIRT1 expression was inhibited, the expression of ZO-1 protein (Figures 6A,B) and mRNA (Figure 6C) in IEC6 cells decreased. After SIRT1 expression was inhibited, VEGF mRNA expression in IEC6 cells decreased (Figure 6D), and VEGF protein expression in cells increased (Figure 6E). There was almost no expression of VEGF protein in the cell culture medium, which was lower than the lower limit of the actual detection box. This confirmed that SIRT1 can regulate the expression of ZO-1 and VEGF to maintain intestinal mucosal barrier function.

The proliferation and migration of intestinal epithelial cells are important factors to maintain the stability of intestinal mucosal barrier function. After inhibiting SIRT1 expression in IEC6 cells, the proliferation ability (Figure 6F) and migration ability (Figures 6G,H) of cells were significantly inhibited, which confirmed that SIRT1 plays a role in maintaining the proliferation and migration ability of epithelial cells and also participates in maintaining the intestinal mucosal barrier function.

### Effect of Sirtuin1 or Silent Mating–Type Information Regulation 2 homolog-1 (SIRT1) on Epithelial–Mesenchymal Transformation (EMT) of Intestinal Epithelial Cells

**FIGURE 7:**
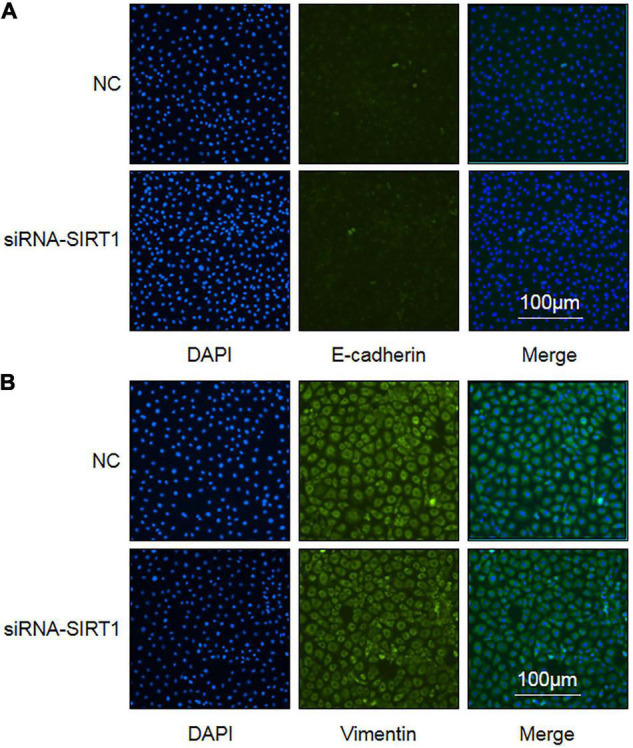
The influence of Sirtuin1 or Silent mating–type information regulation 2 homolog-1 (SIRT1) on the expression of epithelial–mesenchymal transition (EMT) marker proteins in intestinal epithelial cells (IEC). **(A)** Immunofluorescence staining of E-cadherin protein in control negative (NC) groups and siRNA-SIRT1 group cells (10 × 10). **(B)** Immunofluorescence staining of Vimentin protein in NC groups and siRNA-SIRT1 group cells (10 × 10).

Epithelial–mesenchymal transformation is one of the important mechanisms of intestinal fibrosis and is the main source of intestinal fibroblasts. Detecting the expression changes of E-cadherin and Vimentin, the marker proteins of EMT, is helpful to judge the occurrence of EMT. After SIRT1 expression was inhibited, there was no significant difference in the number and fluorescence intensity of E-cadherin (Figure 7A) and Vimentin (Figure 7B) protein-positive expression between the two groups. This suggests that SIRT1 may not be involved in the EMT of intestinal epithelial cells, but this result needs to be confirmed by more relevant experiments.

In summarizing the cytological experiment results, we can preliminarily find that SIRT1 can regulate the expression of fibrosis-related proteins in the intestinal epithelial cells to participate in the occurrence of intestinal fibrosis. SIRT1 can protect the proliferation and migration of intestinal epithelial cells and also can regulate the expression of ZO-1 and VEGF proteins, which are related to maintaining mucosal barrier function.

## Discussion

Intestinal injury caused by inflammatory response is one of the main mechanisms to stimulate the occurrence and development of NEC (12). Inflammation is an important factor in intestinal fibrosis. As a result, inflammatory response may be an important cause of intestinal stenosis in NEC. In the present study, 43 children with enterostricture presented a sustained rise in serum CRP during NEC development, indicating that inflammation is a risk factor for enterostricture secondary to NEC (13). H and E staining images presented obvious fibrous hyperplasia in the intestinal submucosa of the narrow segment. Immunohistochemical results indicated that TGF-β1 protein was overexpressed in narrow intestinal tissues. These pathological phenomena are similar to enterostricture caused by IBD (5, 6), signifying that enterofibrosis caused by inflammation may be the symptom of enterostricture secondary to NEC.

The immunohistochemical results illustrated that SIRT1 protein was less expressed in the mucosal layer and submucosal layer of the intestinal tissue in the narrow segment. The higher the serum CRP level in those NEC patients, the expression of SIRT1 protein was lesser. SIRT1 protein was negatively correlated with TGF-β1 protein expression in narrow intestinal tissues. The possible reason is that the downsized expression of SIRT1 protein in the NEC intestinal tissue leads to the decreased ability of SIRT1 to curb inflammatory response (14). SIRT1 acts to curb inflammatory response and alleviate NEC intestinal injury in the acute phase of NEC (15, 16). Therefore, an unbalanced inflammatory response in the development of NEC may contribute to the cultivation of fibrosis. Intestinal tissue will repair itself when it is injured by acute or chronic inflammation, and fibrosis is a key factor in the self-repair process (17). Intestinal inflammatory damage is the main pathological change of NEC. After NEC is treated, intestinal fibrosis initiates to repair NEC. Combined with the low expression of SIRT1 in NEC secondary intestinal stenosis tissues, it can be initially inferred that SIRT1 may be engaged in the regulation of inflammation and fibrosis.

Judging from some studies regarding renal fibrosis, it has been found that activation of SIRT1 can curb TGF-β1/Smad3 expression to alleviate fibrosis (18). Nonetheless, *in vitro* cell experiments indicated that the expressions of TGF-β1 and Smad3 also decreased after curbing the expression of SIRT1, which were opposite to the results of clinical specimens. The possible reason for such analyzes is that TGF-β1 is a protective factor during the occurrence and development of NEC. TGF-β1 and TGF-β2 in breast milk have protective effects on intestinal epithelial cells in premature infants, which can enhance the proliferation ability of intestinal epithelial cells, enhance the expression of tight junction proteins, such as ZO-1, maintain intestinal mucosal barrier function, and downsize NEC (19, 20). Intestinal stenosis in NEC is the result of the repair of intestinal inflammatory damage in the acute phase of NEC. The clinical treatment of children with secondary intestinal stenosis to NEC is more satisfactory than that of children in the acute phase of NEC. Therefore, the overexpression of TGF-β1 protein in the intestinal tissue of children with NEC secondary intestinal stenosis is not necessarily harmful.

Our study found that the expression of SIRT1 decreased with the progression of Bell’s staging, but the difference was not statistically significant. Analysis of possible reasons for this resulted in bias due to the small sample size. It is also possible that the children with Bell’s Stage I in this study retrospectively analyzed their clinical symptoms to determine the Bell staging, so there is a certain degree of subjectivity and bias. It is also possible that the expression of SIRT1 is also restored to a certain extent during the repair of intestinal injury, leading to biased results. This study also found that the expression of TGF-β1 in the intestinal tissue of children with Bell’s Stage II was elevated. Analysis of the possible reason is that the degree of inflammation and injury of intestinal tissue in children with Bell’s Stage II is more clear and serious. The repair process of damaged intestinal tissue was also more pronounced, and the high degree of fibrosis resulted in increased TGF-β1 expression.

In IEC6 cells, the expressions of TGF-β1, Smad3, and ZO-1 were downsized when SIRT1 expression was curbed, while other studies have found that TGF-β1 and ZO-1 can protect intestinal epithelial cells and intestinal mucosal barrier function during NEC (19, 20). Combined with our findings, we estimate that SIRT1 may be TGF-β1 and ZO-1 upstream gene that can enhance the expression of TGF-β1 and ZO-1 to protect intestinal epithelial cells and intestinal mucosal barrier function. The results of clinical specimen experiments and *in vitro* cell experiments were inconsistent. Thus, we presumed that the low expression of SIRT1 and the overexpression of TGF-β1 might be affected by the overexpressed NF-κB signaling pathway.

Studies indicated that the expression of SIRT1 is downsized in NEC necrotic intestinal tissue, which may be relevant to the activation of TLR4/NF-κB signal pathway in NEC intestinal tissue (16). Toll-like receptor 4 (TLR4) enhances the development of NEC by mediating NF-κB signaling pathway to enhance the expression of tumor necrosis factor-α (TNF-α), interleukin-1 (IL-1), and other inflammatory mediators (21). The expression of SIRT1 protein indicated low in NEC secondary intestinal stenosis, stating that the low expression of SIRT1 may be related to the activation of NF-κB signaling pathway. The protein of NF-κB P65 was significantly raised in fibrotic lung tissues while the pulmonary fibrosis was alleviated after curbing the protein expression of NF-κB P65, indicating that the raised protein content of NF-κB is closely related to fibrosis (22). SIRT1 can modify NF-κB P65 through deacetylation, thus reducing the activity of NF-κB P65 and curbing its transcription. SIRT1 also downsized the release of TNF-α, IL-6, and other inflammatory factors and alleviated the inflammatory response (23). NF-κB also regulates TGF-β1/Smad3 expression to enhance fibrosis (24). In the *in vitro* cell experiments, the expression of NF-κB p65 decreased, and the expression of TNF-α also changed after curbing the expression of SIRT1. This result is contrary to the previous research conclusion, signaling that SIRT1 and NF-κB p65 are related. Through the combination of the two separate results, we analyze the possible reasons for this phenomenon.

Sirtuin1 or Silent mating–type information regulation 2 homolog-1 (SIRT1) and NF-kB are two mutually antagonistic signaling pathways in metabolic and inflammatory mechanisms. SIRT1 can curb the transcriptional activation of NF-κB by deacetylating the Lys310 site of NF-κB P65/RelA, while NF-κB can reversely curb the expression of SIRT1 (23). NF-κB was able to suppress SIRT1 expression by triggering miR34a (23). Reactive oxygen species (ROS) can stimulate and activate the NF-κB signaling pathway to enhance inflammatory responses while NF-κB also can enhance ROS to enhance oxidative stress (25). ROS can oxidize the cysteine residues of SIRT1, enhance the degradation of SIRT1, and curb the expression of SIRT1 (26). NF-κB also enhances inflammatory responses by stimulating interferon-γ (IFN-γ) expression, which curbs SIRT1 expression in skeletal muscle (27). During the acute phase of NEC, inflammatory signaling systems, such as NF-κB, ROS, IFN-γ, IL-6, and TLR4, all raised with their expression (28). These signaling pathways may curb the expression of SIRT1 protein in the process of NEC. Our clinical results indicated that IL-6 and IFN-γ in the blood of children with NEC secondary intestinal stricture were raised to a certain extent during the acute phase of NEC. The expression of SIRT1 protein was lower in intestinal tissue of infants with neonatal respiratory distress syndrome during NEC. Combined with the conclusions of the above literature review, it can be concluded that the reason for the low expression of SIRT1 in NEC secondary intestinal stricture tissue was the curb on of the overexpressed NF-κB signaling pathway.

In theory, NF-κB p65 expression would raise after curbing SIRT1 expression in cells. However, the results of this study illustrated that after curbing SIRT1 expression in IEC6 cells, NF-κB p65 expression also decreased. The reason for such analysis is that NF-κB p65 in intestinal epithelial cells requires a variety of external factors, such as LPS, TNF-α, and hypoxia. NF-κB p65 was inactivated and only reflected protein expression in IEC6. SIRT1 can not only curb the activation of NF-κB p65 through deacetylation but also can stop the migration of NF-κB p65 in the cytoplasm to the nucleus. NF-κB p65 needs to enter the nucleus to be activated to enhance the inflammatory response. The qRT-PCR and WB adopted in this study located the changes of total mRNA and total protein in IEC6 cells, without distinguishing between the nucleus and cytoplasm, and did not detect the activity changes of NF-κB p65. The bias in choosing IEC6 cells to replace the NEC *in vitro* model also may be responsible for this result. Although IEC6 is one of the commonly used *in vitro* model cells for constructing NEC, IEC6 cannot fully reflect the real situation of human NEC in some functions and mechanisms (29). This mechanism requires other NEC models for an explanation.

The results show that the TNF was not elevated during acute NEC. Many studies found that the low expression of SIRT1 led to increased acetylation of NF-κB p65 in colitis models and promoted the inflammatory response. However, SIRT1 may not regulate the expression of TNF-α; on the contrary, overexpressed TNF-α can inhibit the expression of SIRT1 (30). The *in vitro* experimental data show that the TNF-a mRNA increased in SIRT1 knockdown (Figure 4), but the protein levels decreased (Figure 5). We hypothesized that SIRT1 may not be involved in the direct regulation of TNF-α in post-NEC intestinal stricture. This conclusion needs further verification.

Intestinal epithelial barrier dysplasia is an important mechanism in preterm infants prone to NEC. The proliferation and apoptosis, migration ability, and tight protein linkage of intestinal epithelial cells are important factors for maintaining the stable state of the epithelial cells. When NEC occurs, intestinal epithelial cells raise apoptosis, decrease proliferation ability, raise permeability, and decrease tight junction protein ZO-1 protein expression (31). After the homeostasis of intestinal epithelial cells is destroyed, the barrier dysfunction can result in intestinal damage while the repair of tissue damage is also an important process of fibrosis. Therefore, intestinal epithelial barrier dysplasia may be an important mechanism of NEC secondary intestinal stricture. SIRT1 is vital in maintaining the homeostasis of intestinal epithelial cells. Downsized SIRT1 expression can aggravate the occurrence of colitis (32). In this study, it was found that the proliferation and migration ability of cells was significantly weakened while the expression of ZO-1 protein was downsized after SIRT1 expression was curbed in IEC-6 cells, signifying that SIRT1 could enhance the proliferation and migration ability of intestinal epithelial cells and the expression of ZO-1 to maintain the homeostasis of intestinal epithelial cells and to protect the barrier function to downsize the damage of NEC. This may also be the mechanism that SIRT1 participates in the occurrence of NEC secondary intestinal stenosis.

The growth factor of vascular endothelial is highly expressed in pulmonary fibrosis, which is positively correlated with the degree of pulmonary fibrosis (33). In intestinal fibrosis, VEGF not only enhances the transformation of intestinal epithelial cells into mesenchymal cells and stimulates the secretion of fibrinogen by fibroblasts to aggravate intestinal fibrosis, VEGF also enhances the formation of microvessels and plays a role in the repair of intestinal injury to aggravate intestinal fibrosis (34). However, in renal fibrosis, VEGF can improve renal fibrosis by triggering vascular remodeling and reducing inflammation (35, 36). These studies demonstrate that VEGF is controversial in the mechanism of fibrosis. Research studies have found that VEGF is downsized in the development of NEC. This may be caused by prenatal inflammation leading to intestinal injury and downsized VEGF expression. Increased VEGF expression can enhance the repair of intestinal injury (37). Our research found that after SIRT1 expression was curbed in IEC-6 cells, the protein expression level of intracellular VEGF raised, but the mRNA expression of VEGF decreased. This signifies that SIRT1 can regulate VEGF protein expression in IEC-6 cells to participate in NEC, but the specific mechanism needs further explanation. The expression level of VEGF protein in the IEC-6 cell culture medium of the control group and sirNA-SIRT1 group was lower than the lower limit detected by the kit, indicating that intestinal epithelial cells hardly secreted VEGF protein. Epithelial–mesenchymal transformation (EMT) is another important process in the pathogenesis of intestinal fibrosis. It is the major source of intestinal fibroblasts. Research on the invasion and migration of lung cancer cells found that resveratrol, a SIRT1 agonist, could curb the occurrence of EMT to downsize the invasion and migration of lung cancer cells. The mechanism was related to SIRT1 regulating the expression of E-cadherin, a marker of EMT (38). It has also been found in renal fibrosis research studies that raised SIRT1 expression can curb TGF-β1 and Smad3 expression to downsize the occurrence of EMT (8). Our research found that the expression of E-cadherin and Vimentin did not change significantly after the expression of SIRT1 was curbed in IEC-6 cells, showing that SIRT1 may have no significant effect on the occurrence of EMT in IEC-6 cells. The possible reason is that EMT is not involved in the occurrence of NEC secondary intestinal stenosis, which needs to be confirmed by further studies.

## Conclusion

Compared with the prognosis of children with NEC in the acute stage, the occurrence of intestinal stricture in NEC is not necessarily a bad outcome. It is the outcome of the repair of intestinal injury in NEC. In a sense, NF-kB p65 can enhance the expression of TGF-β1 and Smad3 to enhance intestinal fibrosis and also may be beneficial to the outcome of NEC. Therefore, we deduce that the role of SIRT1 in NEC secondary intestinal stricture is positive. On the one hand, SIRT1 can protect the proliferation and migration of intestinal epithelial cell and can enhance the expression of TGF-β1 and ZO-1 to protect intestinal epithelial cells. On the other hand, the low expression of SIRT1 lost the ability to curb NF-κB p65 so that the activation of NF-κB signaling pathway enhanced the occurrence of inflammatory response and intestinal fibrosis, and elevated the transition of NEC from the acute phase to the chronic phase. However, the specific mechanism of SIRT1 in NEC intestinal tissue still needs verification. The role of SIRT1 and NF-κB, TGF-β1, and other signaling pathways is the network, not point-to-point. The activity state of SIRT1 also depends on cells or the environment, on stage, or state of the disease.

Sirtuin1 or Silent mating–type information regulation 2 homolog-1 (SIRT1) and TGF-β1 are playing a role in suppressing inflammation in NEC. The mechanism may be that SIRT1 promotes the expression of TGF-β1 to inhibit the inflammatory response and promote the repair of intestinal epithelial cell damage. Reduced expression of SIRT1 in NEC children’s intestinal epithelium can result in a reduced ability of SIRT1 to promote TGF-β1 expression and aggravated inflammatory damage. On the other hand, the expression of TGF-β1 is regulated by a variety of signaling pathways, and the expression level increases during the process of intestinal inflammatory injury repair and development of intestinal fibrosis. Studies in ulcerative colitis have found that promoting the expression of TGF-β1 can repair damaged bowel and promote fibrosis progression. It is speculated that the elevated expression of TGF-β1 may be the result rather than the origin of intestinal fibrosis. Therefore, the signaling pathway that SIRT1 regulates TGF-β1 in NEC may depend on the immune status of intestinal tissue. The exact mechanism and balance of SIRT1’s regulation of TGF-β1 during NEC need to be further studied.

This research found the expression characteristics of SIRT1 in NEC intestinal stenosis and initially analyzed the possible mechanism of SIRT1. The results are innovative, which could expand the related research in the field of NEC, and help to further find the prevention or improvement measures of NEC secondary intestinal stenosis. However, the limitations of this research include a small number of clinical trial specimens, lack of immunohistochemical results of other fibrosis indicators, no NEC-related *in vitro* cell model, and no SIRT1 overexpressed cell model. Therefore, the experimental conclusions shall be finalized with further data and clinical figures. Animal experiments and more cytological experiments are needed to fully explain the specific regulatory mechanism of SIRT1 on NEC secondary intestinal stenosis.

## Data Availability Statement

The original contributions presented in this study are included in the article/Supplementary Material, further inquiries can be directed to the corresponding author.

## Ethics Statement

The studies involving human participants were reviewed and approved by Ethics Committee of The Children’s Hospital Zhejiang University School of Medicine. Ethical review number: 2021-IRB-048. Written informed consent to participate in this study was provided by the participants or their legal guardian/next of kin.

## Author Contributions

RC and CL: conception and design, collection and/or assembly of data, data analysis and interpretation, manuscript writing, and final approval of manuscript. YZ, WG, LZ, and BS: collection and/or assembly of data, data analysis, and interpretation. JT: conception and design and final approval of manuscript. All authors contributed to the article and approved the submitted version.

## Conflict of Interest

The authors declare that the research was conducted in the absence of any commercial or financial relationships that could be construed as a potential conflict of interest.

## Publisher’s Note

All claims expressed in this article are solely those of the authors and do not necessarily represent those of their affiliated organizations, or those of the publisher, the editors and the reviewers. Any product that may be evaluated in this article, or claim that may be made by its manufacturer, is not guaranteed or endorsed by the publisher.

## Supplementary Material

Supplementary Figure 1 The expression of Sirtuin1 or Silent mating–type information regulation 2 homolog-1 (SIRT1) and transforming growth factor-β1 (TGF-β1) in post-necrotizing enterocolitis (NEC) intestinal stricture tissues (more images).

## References

[1] Neu J Walker WA. Necrotizing enterocolitis. *N Engl J Med.* (2011) 364:255–64. 10.1056/NEJMra1005408 21247316 PMC3628622

[2] Meister AL Doheny KK Travagli RA. Necrotizing enterocolitis: it’s not all in the gut. *Exp Biol Med (Maywood).* (2020) 245:85–95. 10.1177/1535370219891971 31810384 PMC7016421

[3] Burnand KM Zaparackaite I Lahiri RP Parsons G Farrugia MK Clarke SA The value of contrast studies in the evaluation of bowel strictures after necrotising enterocolitis. *Pediatr Surg Int.* (2016) 32:465–70. 10.1007/s00383-016-3880-7 26915085

[4] Janik JS Ein SH Mancer K. Intestinal stricture after necrotizing enterocolitis. *J Pediatr Surg.* (1981) 16:438–43. 10.1016/s0022-3468(81)80002-4 7277135

[5] Yang B Zhang G Elias M Zhu Y Wang J. The role of cytokine and immune responses in intestinal fibrosis. *J Dig Dis.* (2020) 21:308–14. 10.1111/1751-2980.12879 32410365

[6] Graham MF Bryson GR Diegelmann RF. Transforming growth factor beta 1 selectively augments collagen synthesis by human intestinal smooth muscle cells. *Gastroenterology.* (1990) 99:447–53. 10.1016/0016-5085(90)91028-5 2365193

[7] Ramirez T Li Y Yin S Xu M Feng D Zhou Z Aging aggravates alcoholic liver injury and fibrosis in mice by downregulating Sirtuin 1 expression. *J Hepatol.* (2017) 66:601–9. 10.1016/j.jhep.2016.11.004 27871879 PMC5316497

[8] Kong L Wu H Zhou W Luo M Tan Y Miao L Sirtuin 1: a target for kidney diseases. *Mol Med.* (2015) 21:87–97. 10.2119/molmed.2014.00211 25587857 PMC4461580

[9] Sweet DG Carnielli V Greisen G Hallman M Ozek E Pas AT European consensus guidelines on the management of respiratory distress syndrome – 2019 update. *Neonatology.* (2019) 115:432–50. 10.1159/000499361 30974433 PMC6604659

[10] Lai D Tang J Chen L Fan EK Scott MJ Li Y Group 2 innate lymphoid cells protect lung endothelial cells from pyroptosis in sepsis. *Cell Death Dis.* (2018) 9:369. 10.1038/s41419-018-0412-5 29511181 PMC5840374

[11] Schmittgen TD Livak KJ. Analyzing real-time PCR data by the comparative C(T) method. *Nat Protoc.* (2008) 3:1101–8. 10.1038/nprot.2008.73 18546601

[12] Lin PW Stoll BJ. Necrotising enterocolitis. *Lancet.* (2006) 368:1271–83. 10.1016/S0140-6736(06)69525-1 17027734

[13] Zhang H Chen J Wang Y Deng C Li L Guo C. Predictive factors and clinical practice profile for strictures post-necrotising enterocolitis. *Medicine (Baltimore).* (2017) 96:e6273. 10.1097/MD.0000000000006273 28272242 PMC5348190

[14] Bai M Lu C An L Gao Q Xie W Miao F SIRT1 relieves necrotizing enterocolitis through inactivation of hypoxia-inducible factor (HIF)-1a. *Cell Cycle.* (2020) 19:2018–27. 10.1080/15384101.2020.1788251 32657204 PMC7469541

[15] Ma F Hao H Gao X Cai Y Zhou J Liang P Melatonin ameliorates necrotizing enterocolitis by preventing Th17/Treg imbalance through activation of the AMPK/SIRT1 pathway. *Theranostics.* (2020) 10:7730–46. 10.7150/thno.45862 32685016 PMC7359097

[16] Yin Y Wu X Peng B Zou H Li S Wang J Curcumin improves necrotising microscopic colitis and cell pyroptosis by activating SIRT1/NRF2 and inhibiting the TLR4 signalling pathway in newborn rats. *Innate Immun.* (2020) 26:609–17. 10.1177/1753425920933656 32924710 PMC7556186

[17] Bell PD Rockey DC Hill JA. Fibrosis – a common pathway to organ injury and failure. *N Engl J Med.* (2015) 372:1138–49. 10.1056/NEJMra1300575 25785971

[18] Huang X Wen D Zhang M Xie Q Ma L Guan Y SIRT1 activation ameliorates renal fibrosis by inhibiting the TGF-beta/Smad3 pathway. *J Cell Biochem.* (2014) 115:996–1005. 10.1002/jcb.24748 24356887

[19] Shiou SR Yu Y Guo Y Westerhoff M Lu L Petrof EO Oral administration of transforming growth factor-β1 (TGF-β1) protects the immature gut from injury via Smad protein-dependent suppression of epithelial nuclear factor κB (NF-κB) signaling and proinflammatory cytokine production. *J Biol Chem.* (2013) 288:34757–66. 10.1074/jbc.M113.503946 24129565 PMC3843089

[20] Abdelhamid AE Chuang SL Hayes P Fell JME. In vitro cow’s milk protein-specific inflammatory and regulatory cytokine responses in preterm infants with necrotizing enterocolitis and sepsis. *Pediatr Res.* (2011) 69:165–9. 10.1203/PDR.0b013e31820263e7 20975616

[21] Hou Y Lu X Zhang Y. IRAK inhibitor protects the intestinal tract of necrotizing enterocolitis by inhibiting the toll-like receptor (TLR) inflammatory signaling pathway in rats. *Med Sci Monit.* (2018) 24:3366–73. 10.12659/MSM.910327 29784900 PMC5992962

[22] Tian B Patrikeev I Ochoa L Vargas G Belanger KK Litvinov J NF-κB mediates mesenchymal transition, remodeling, and pulmonary fibrosis in response to chronic inflammation by viral RNA patterns. *Am J Respir Cell Mol Biol.* (2017) 56:506–20. 10.1165/rcmb.2016-0259OC 27911568 PMC5449514

[23] Kauppinen A Suuronen T Ojala J Kaarniranta K Salminen A. Antagonistic crosstalk between NF-kappaB and SIRT1 in the regulation of inflammation and metabolic disorders. *Cell Signal.* (2013) 25:1939–48. 10.1016/j.cellsig.2013.06.007 23770291

[24] Zheng Z Zhu W Lei L Liu XQ Wu YG. Wogonin ameliorates renal inflammation and fibrosis by inhibiting NF-kappaB and TGF-beta1/Smad3 signaling pathways in diabetic nephropathy. *Drug Des Devel Ther.* (2020) 14:4135–48. 10.2147/DDDT.S274256 33116403 PMC7549498

[25] Korbecki J Bobiński R Dutka M. Self-regulation of the inflammatory response by peroxisome proliferator-activated receptors. *Inflamm Res.* (2019) 68:443–58. 10.1007/s00011-019-01231-1 30927048 PMC6517359

[26] Thirupathi A Souza CT. Multi-regulatory network of ROS: the interconnection of ROS, PGC-1 alpha, and AMPK-SIRT1 during exercise. *J Physiol Biochem.* (2017) 73:487–94. 10.1007/s13105-017-0576-y 28707280

[27] Li P Zhao Y Wu X Xia M Fang M Iwasaki Y Interferon gamma (IFN-γ) disrupts energy expenditure and metabolic homeostasis by suppressing SIRT1 transcription. *Nucleic Acids Res.* (2012) 40:1609–20. 10.1093/nar/gkr984 22064865 PMC3287208

[28] Agakidou E Agakidis C Gika H Sarafidis K. Emerging biomarkers for prediction and early diagnosis of necrotizing enterocolitis in the era of metabolomics and proteomics. *Front Pediatr.* (2020) 8:602255. 10.3389/fped.2020.602255 33425815 PMC7793899

[29] Fazio LD Beghetti I Bertuccio SN Marsico C Martini S Masetti R Necrotizing enterocolitis: overview on in vitro models. *Int J Mol Sci.* (2021) 22:6761. 10.3390/ijms22136761 34201786 PMC8268427

[30] Devi K Singh N Jaggi AS. Dual role of sirtuin 1 in inflammatory bowel disease. *Immunopharmacol Immunotoxicol.* (2020) 42:385–91. 10.1080/08923973.2020.1790595 32619377

[31] Burge K Bergner E Gunasekaran A Eckert J Chaaban H. The role of glycosaminoglycans in protection from neonatal necrotizing enterocolitis: a narrative review. *Nutrients.* (2020) 12:546. 10.3390/nu12020546 32093194 PMC7071410

[32] Wellman AS Metukuri MR Kazgan N Xu X Xu Q Ren NSX Intestinal epithelial sirtuin 1 regulates intestinal inflammation during aging in mice by altering the intestinal microbiota. *Gastroenterology.* (2017) 153:772–86. 10.1053/j.gastro.2017.05.022 28552621 PMC5581719

[33] Kulkarni YM Dutta S Iyer AK Venkatadri R Kaushik V Ramesh V A proteomics approach to identifying key protein targets involved in VEGF inhibitor mediated attenuation of bleomycin-induced pulmonary fibrosis. *Proteomics.* (2016) 16:33–46. 10.1002/pmic.201500171 26425798 PMC4703486

[34] Lawrance IC Rogler G Bamias G Breynaert C Florholmen J Pellino G Cellular and molecular mediators of intestinal fibrosis. *J Crohns Colitis.* (2017) 11:1491–503. 10.1016/j.crohns.2014.09.008 25306501 PMC5885809

[35] Guise E Engel JE Williams ML Mahdi F Bidwell GL III Chade AR. Biopolymer-delivered vascular endothelial growth factor improves renal outcomes following revascularization. *Am J Physiol Renal Physiol.* (2019) 316:F1016–25. 10.1152/ajprenal.00607.2018 30892933 PMC6580255

[36] Engel JE Williams E Williams ML Bidwell GL III Chade AR. Targeted VEGF therapy induces long-term renal recovery in chronic kidney disease via macrophage polarization. *Hypertension.* (2019) 74:1113–23. 10.1161/HYPERTENSIONAHA.119.13469 31542966 PMC6785403

[37] Yan X Managlia E Tan XD De Plaen IG. Prenatal inflammation impairs intestinal microvascular development through a TNF-dependent mechanism and predisposes newborn mice to necrotizing enterocolitis. *Am J Physiol Gastrointest Liver Physiol.* (2019) 317:G57–66. 10.1152/ajpgi.00332.2018 31125264 PMC6689733

[38] Cha BK Kim YS Hwang KE Cho KH Oh SH Kim BR Celecoxib and sulindac inhibit TGF-β1-induced epithelial-mesenchymal transition and suppress lung cancer migration and invasion via downregulation of sirtuin 1. *Oncotarget.* (2016) 7:57213–27. 10.18632/oncotarget.11127 27528025 PMC5302984

