# Supplementary material for: Expression and Possible Role of Silent Mating Type Information Regulation 2 Homolog 1 in Post-necrotizing Enterocolitis Stricture in vivo and in vitro
Source: Front Pediatr. 2022 Jul 25;10:836128. doi: 10.3389/fped.2022.836128 (PMC9357903; doi:10.3389/fped.2022.836128)

Supplements Fig1 The expression of SIRT1 and TGF-β1 in post-NEC intestinal stricture tissues (more images)

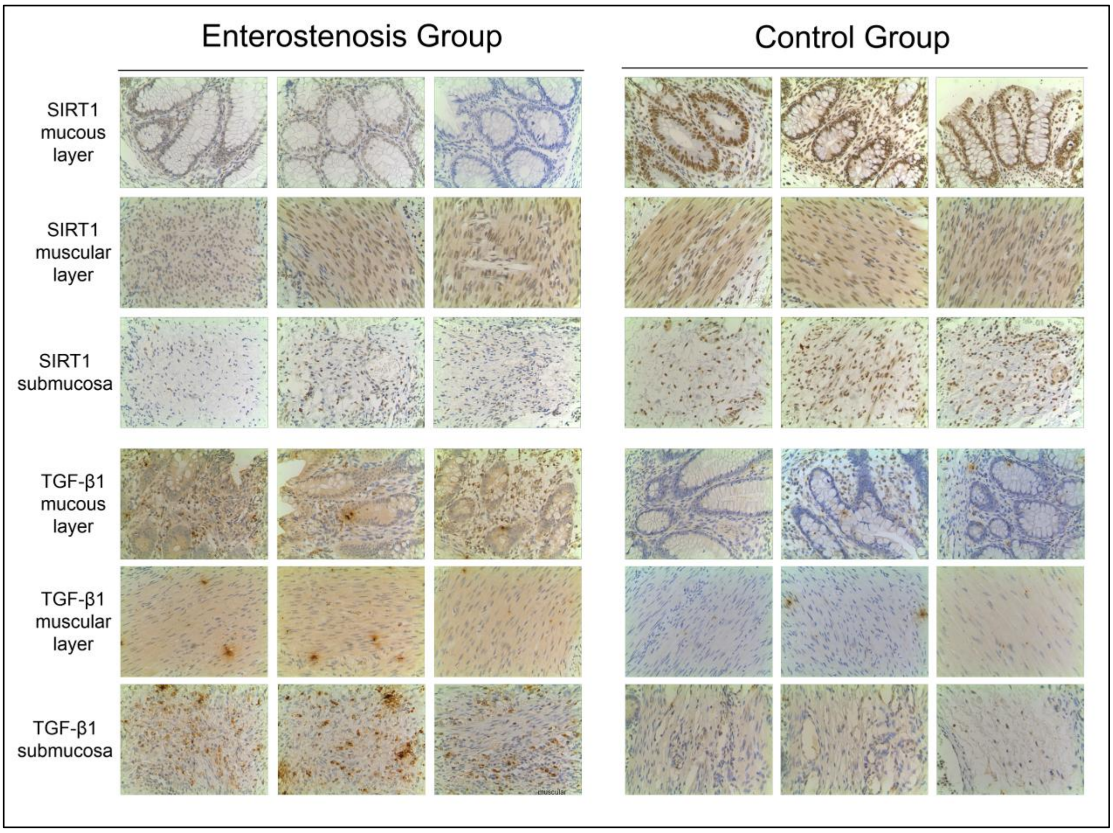

Supplement: Supplementary Figure 1 — The expression of Sirtuin1 or Silent mating–type information regulation 2 homolog-1 (SIRT1) and transforming growth factor-β1 (TGF-β1) in post-necrotizing enterocolitis (NEC) intestinal stricture tissues (more images). [file Image_1.PDF]
