# Supplementary figures and images for: Expression and Possible Role of Silent Mating Type Information Regulation 2 Homolog 1 in Post-necrotizing Enterocolitis Stricture in vivo and in vitro
Source: Front Pediatr. 2022 Jul 25;10:836128. doi: 10.3389/fped.2022.836128 (PMC9357903; doi:10.3389/fped.2022.836128)

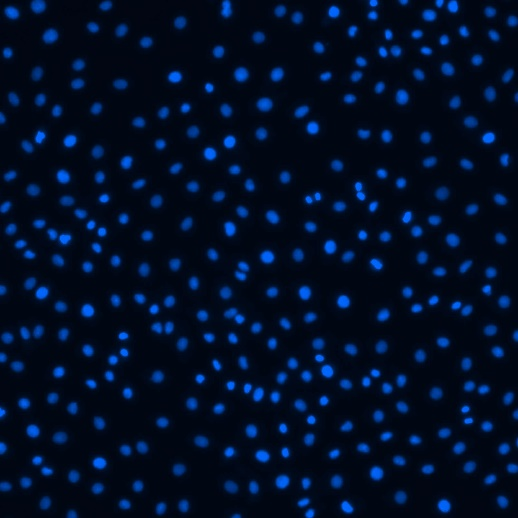

Supplement: Supplementary file 2 [file Data_Sheet_1.ZIP › EMT/E-cadherin/NC dapi.png]

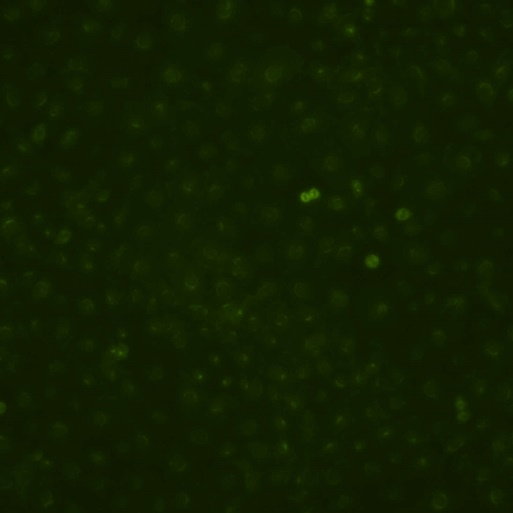

Supplement: Supplementary file 2 [file Data_Sheet_1.ZIP › EMT/E-cadherin/NC.png]

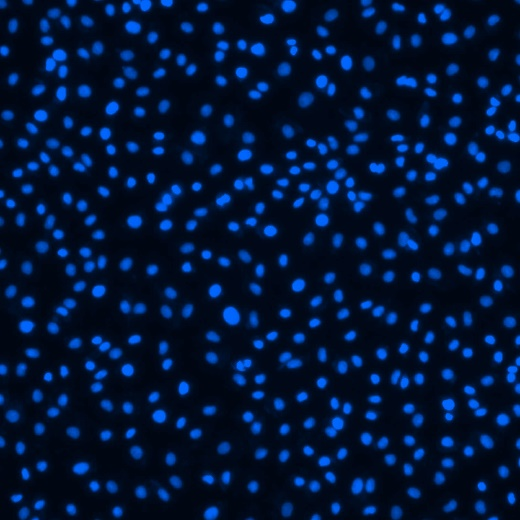

Supplement: Supplementary file 2 [file Data_Sheet_1.ZIP › EMT/E-cadherin/siRNA-SIRT1 dapi.png]

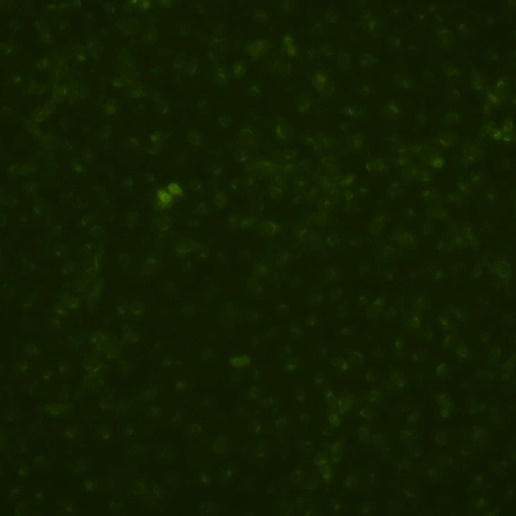

Supplement: Supplementary file 2 [file Data_Sheet_1.ZIP › EMT/E-cadherin/siRNA-SIRT1.png]

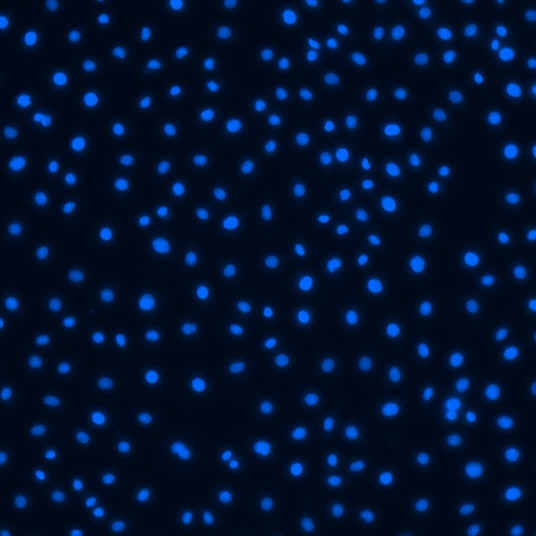

Supplement: Supplementary file 2 [file Data_Sheet_1.ZIP › EMT/Vimentin/NC dapi.png]

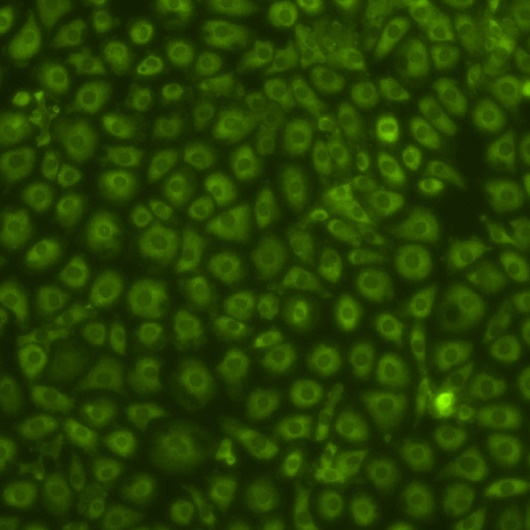

Supplement: Supplementary file 2 [file Data_Sheet_1.ZIP › EMT/Vimentin/NC.png]

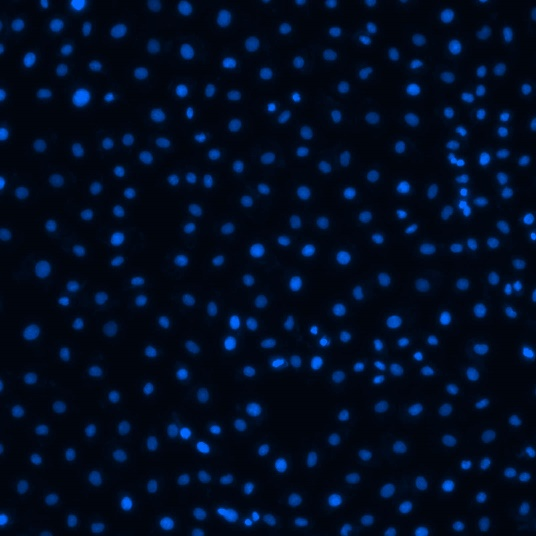

Supplement: Supplementary file 2 [file Data_Sheet_1.ZIP › EMT/Vimentin/siRNA-SIRT1 dapi.png]

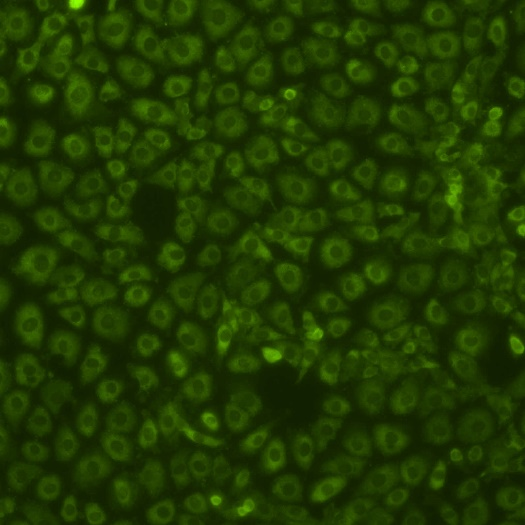

Supplement: Supplementary file 2 [file Data_Sheet_1.ZIP › EMT/Vimentin/siRNA-SIRT1.png]

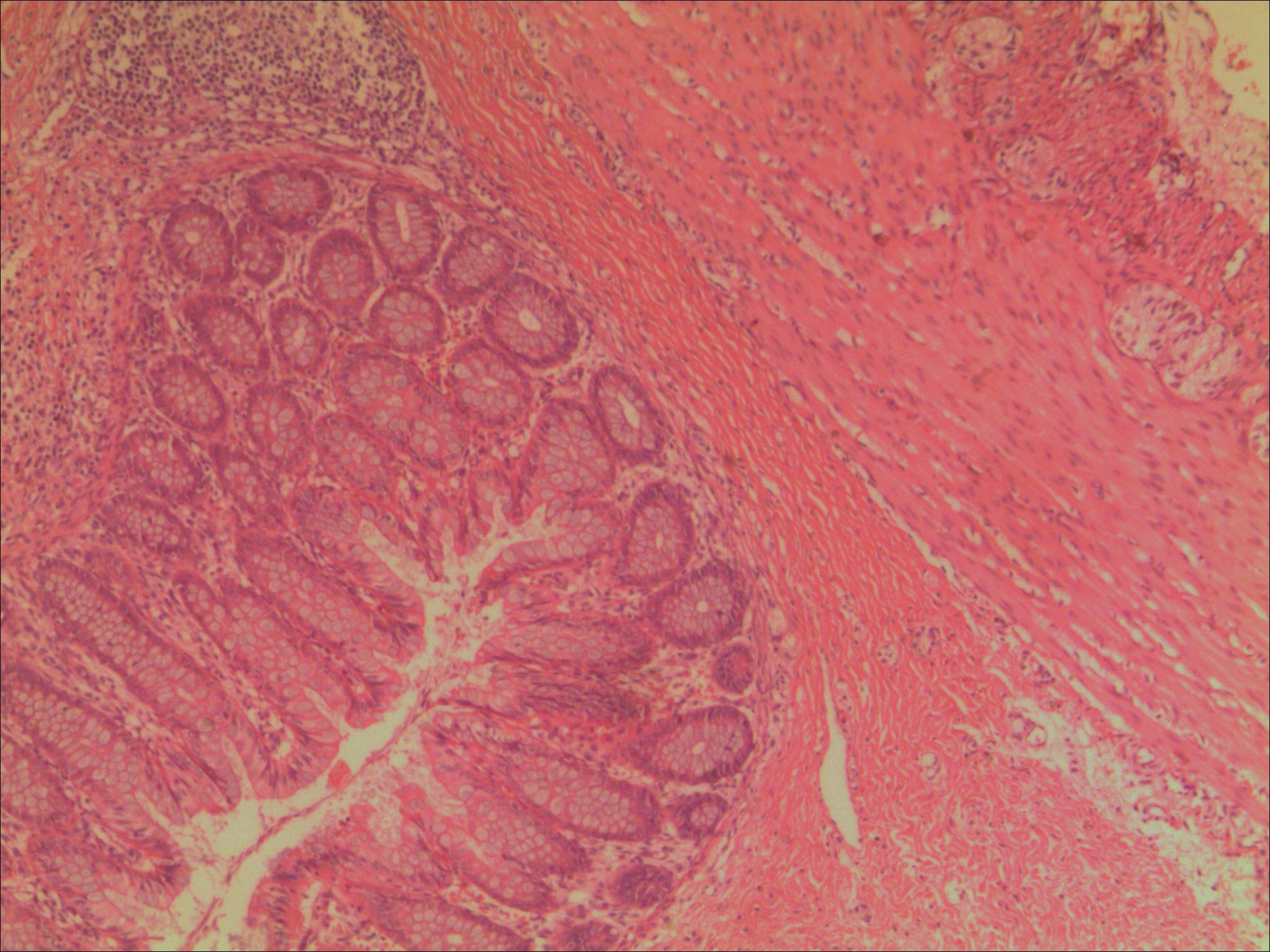

Supplement: Supplementary file 2 [file Data_Sheet_1.ZIP › HE/Intestinal stenosis H-E Staining 5í┴10.jpg]

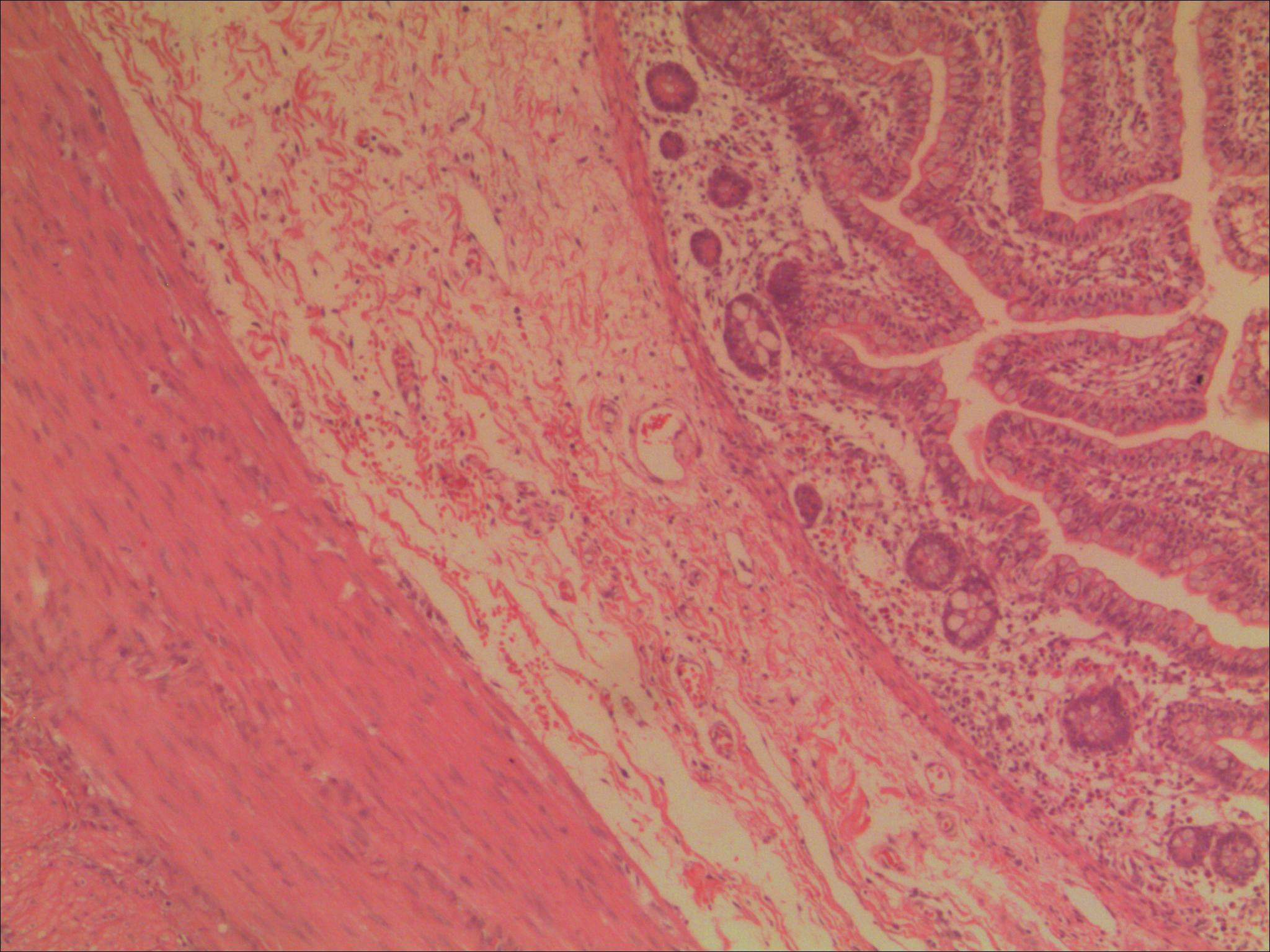

Supplement: Supplementary file 2 [file Data_Sheet_1.ZIP › HE/Normal loops H-E Staining 5í┴10.jpg]

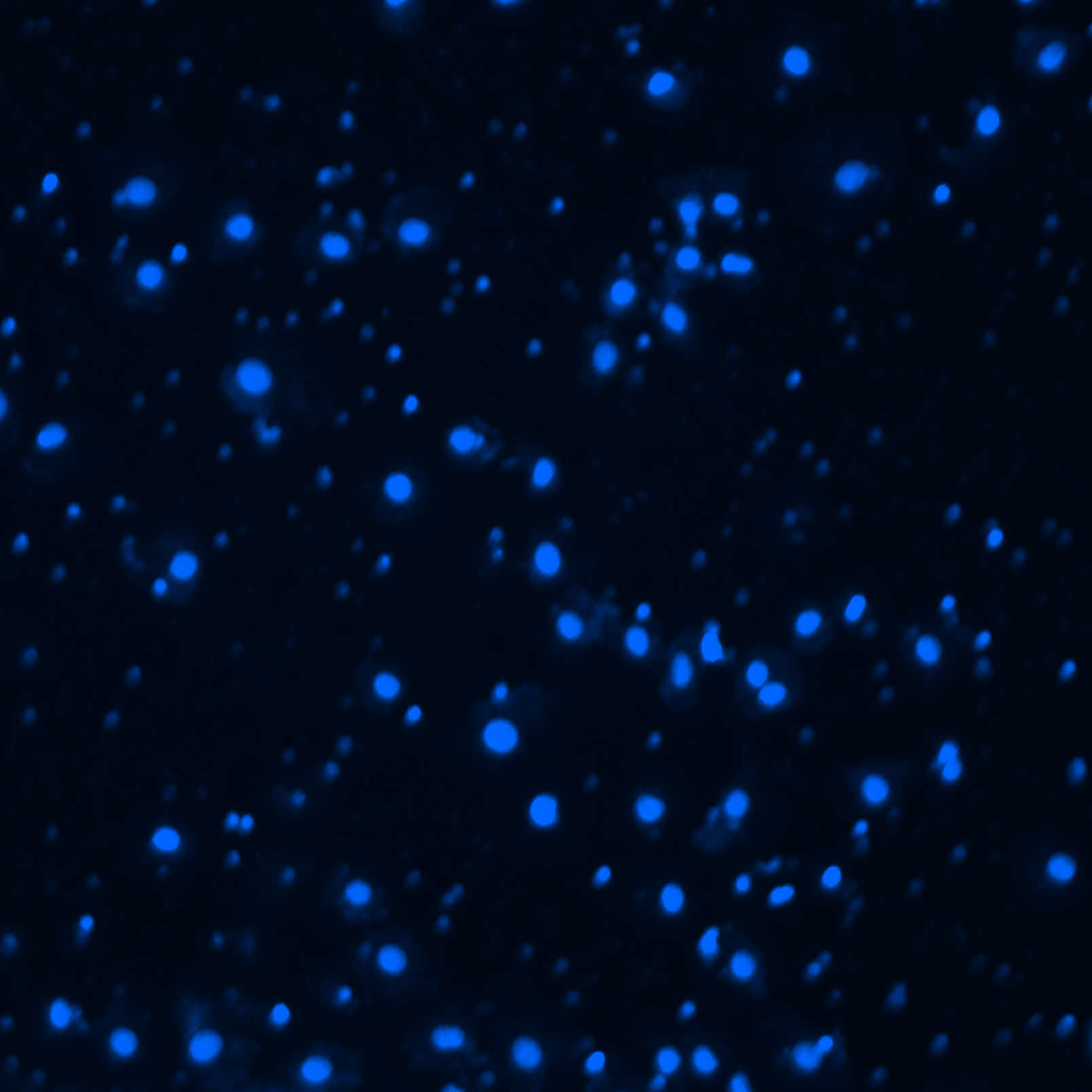

Supplement: Supplementary file 2 [file Data_Sheet_1.ZIP › Transwell/NC/1.jpg]

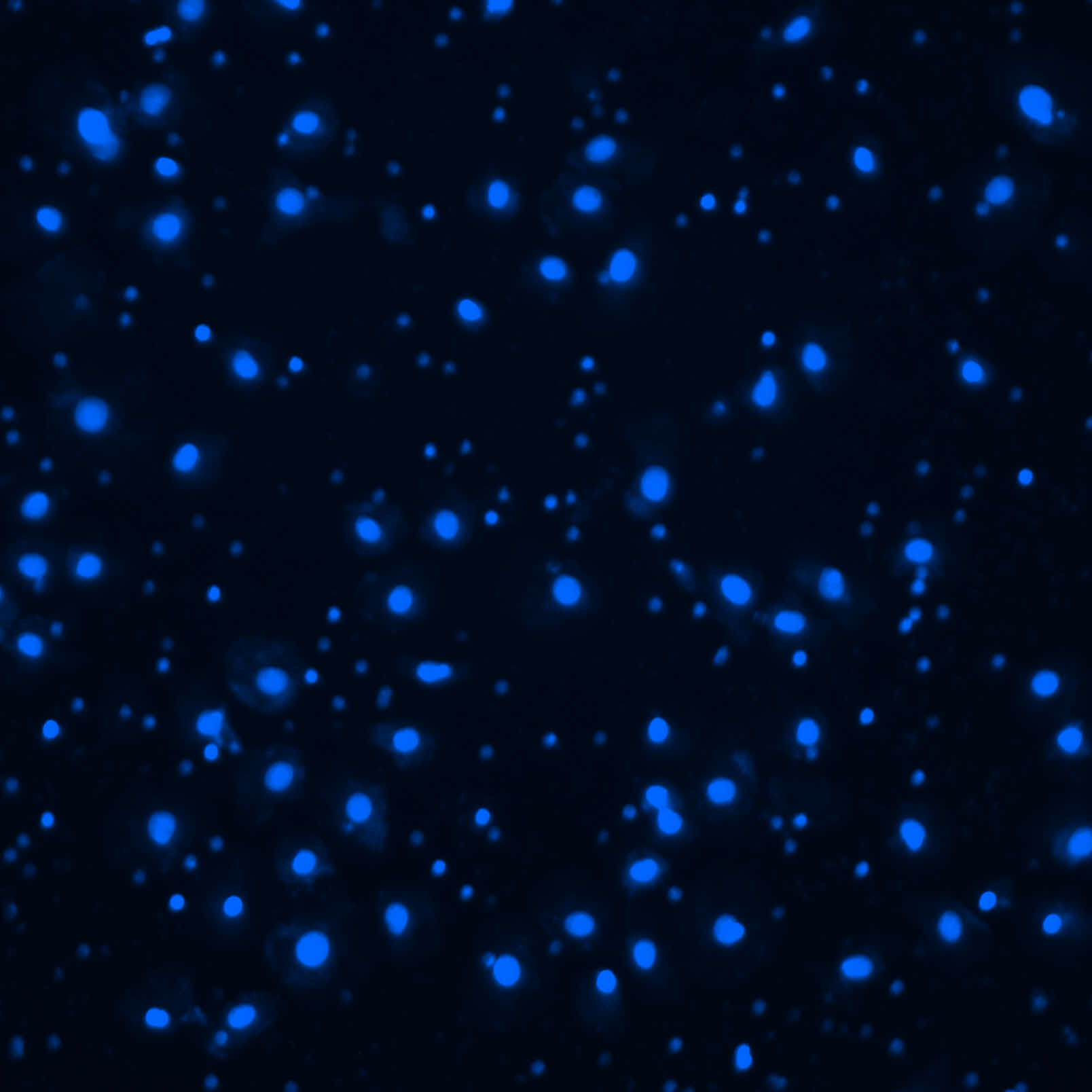

Supplement: Supplementary file 2 [file Data_Sheet_1.ZIP › Transwell/NC/2.jpg]

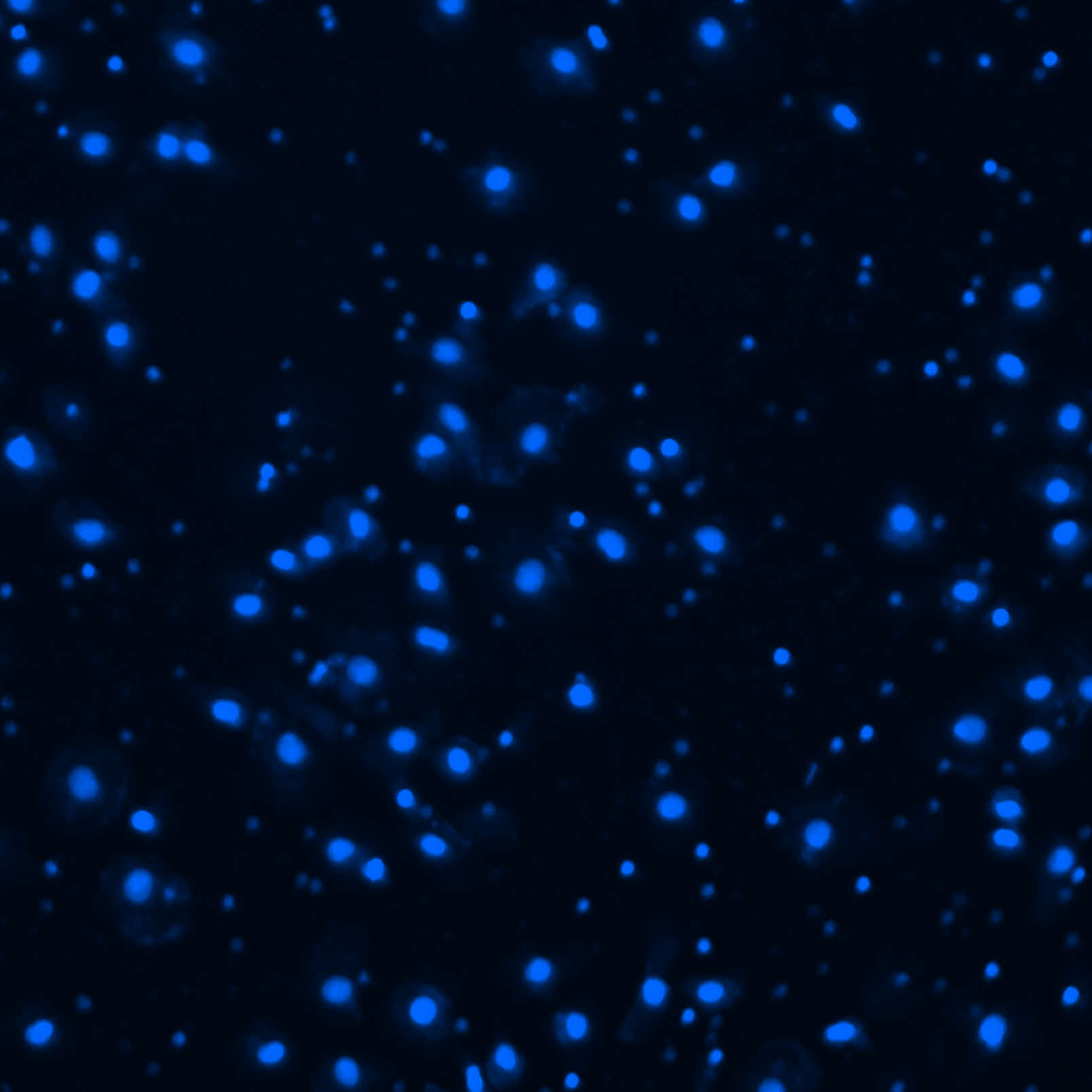

Supplement: Supplementary file 2 [file Data_Sheet_1.ZIP › Transwell/NC/3.jpg]

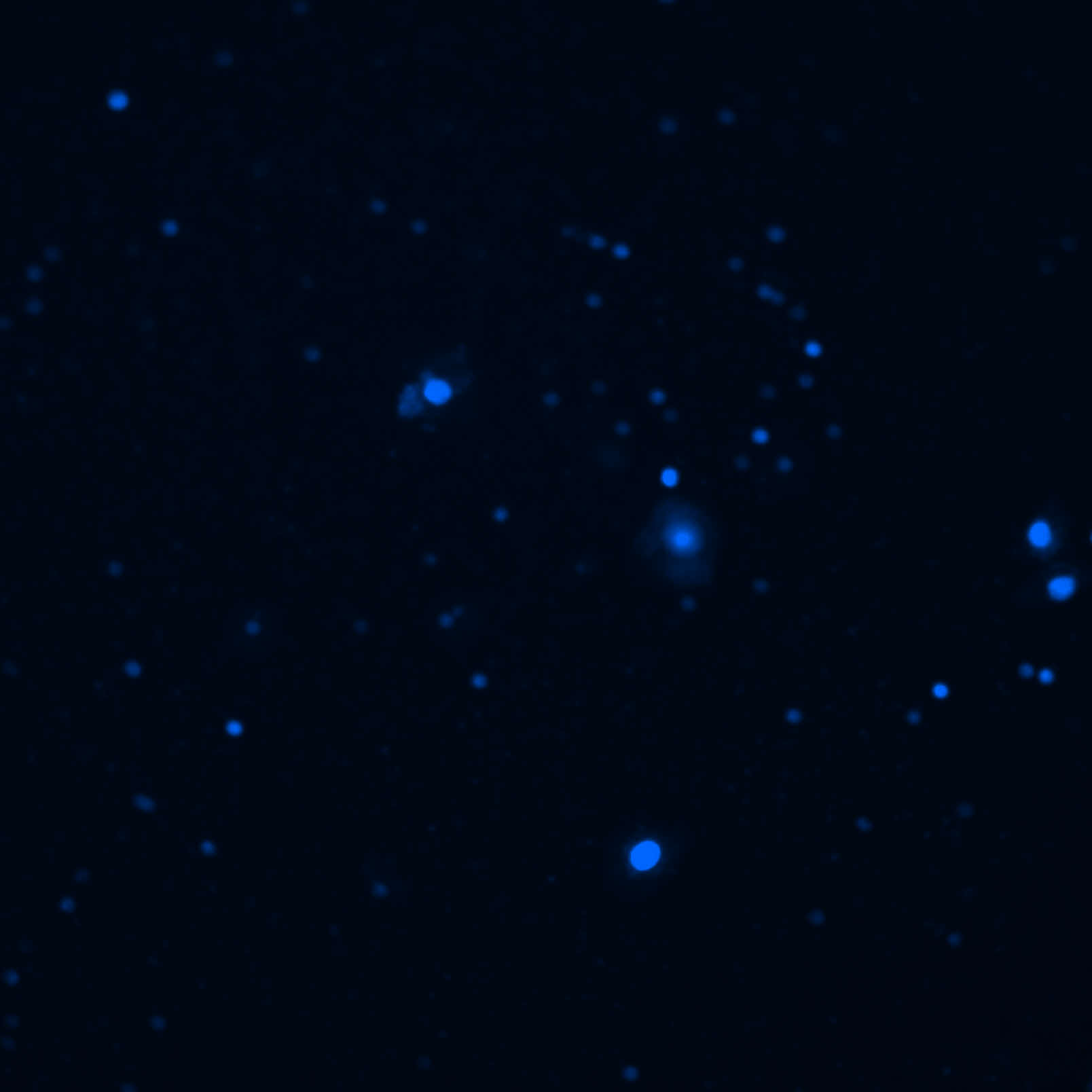

Supplement: Supplementary file 2 [file Data_Sheet_1.ZIP › Transwell/siRNA-SIRT1/1.jpg]

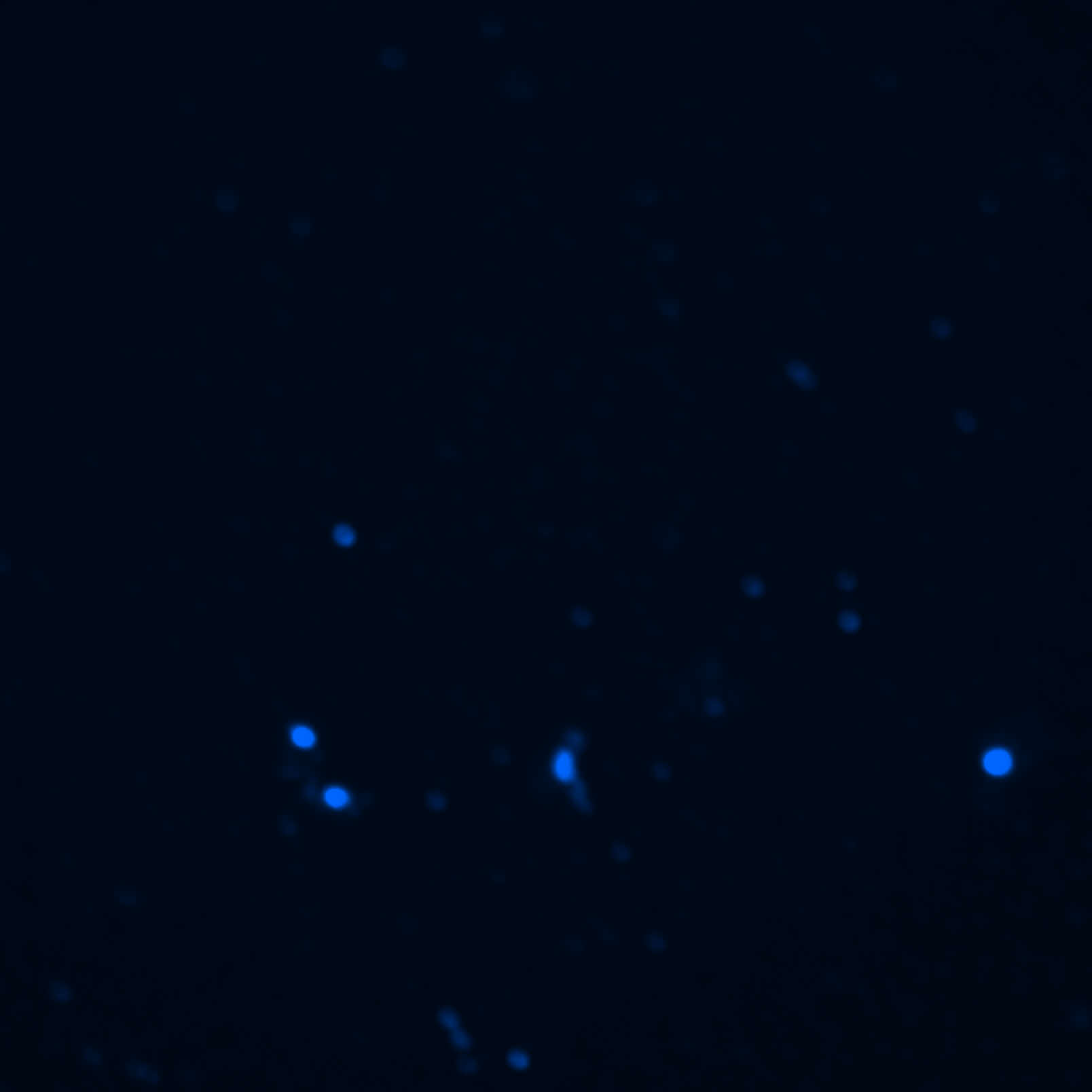

Supplement: Supplementary file 2 [file Data_Sheet_1.ZIP › Transwell/siRNA-SIRT1/2.jpg]

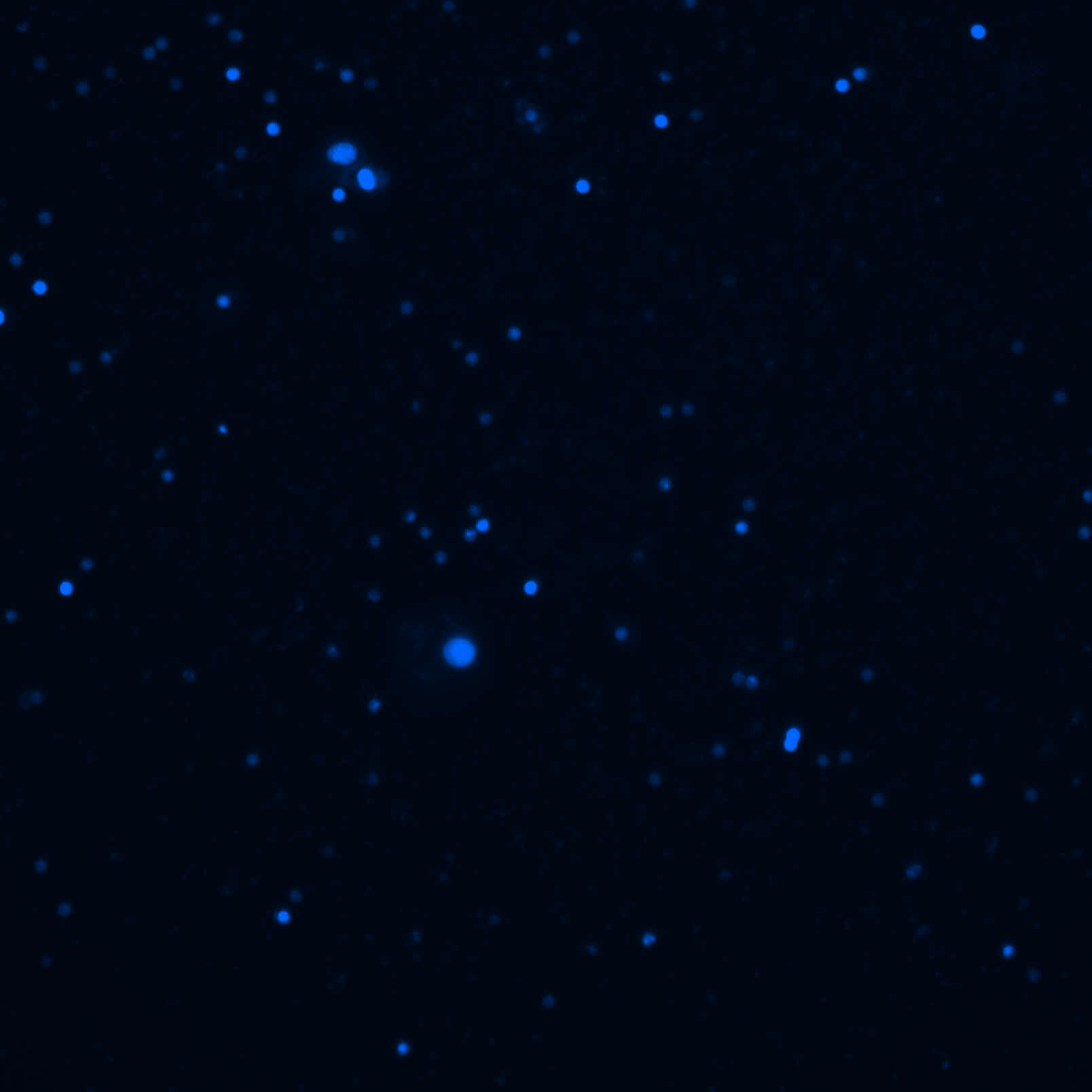

Supplement: Supplementary file 2 [file Data_Sheet_1.ZIP › Transwell/siRNA-SIRT1/3.jpg]

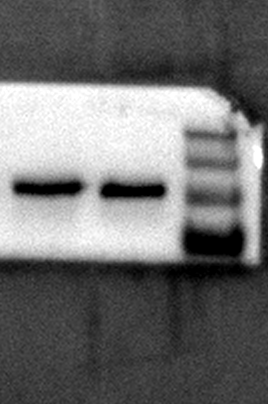

Supplement: Supplementary file 2 [file Data_Sheet_1.ZIP › Western Blot/Film/GAPDH(2).tif]

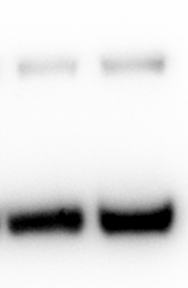

Supplement: Supplementary file 2 [file Data_Sheet_1.ZIP › Western Blot/Film/GAPDH.tif]

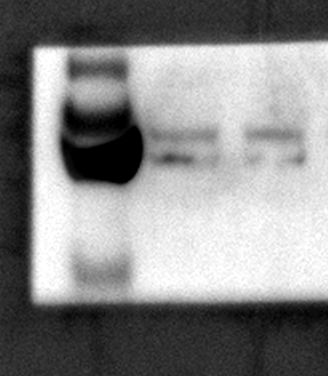

Supplement: Supplementary file 2 [file Data_Sheet_1.ZIP › Western Blot/Film/NF-KB p65.tif]

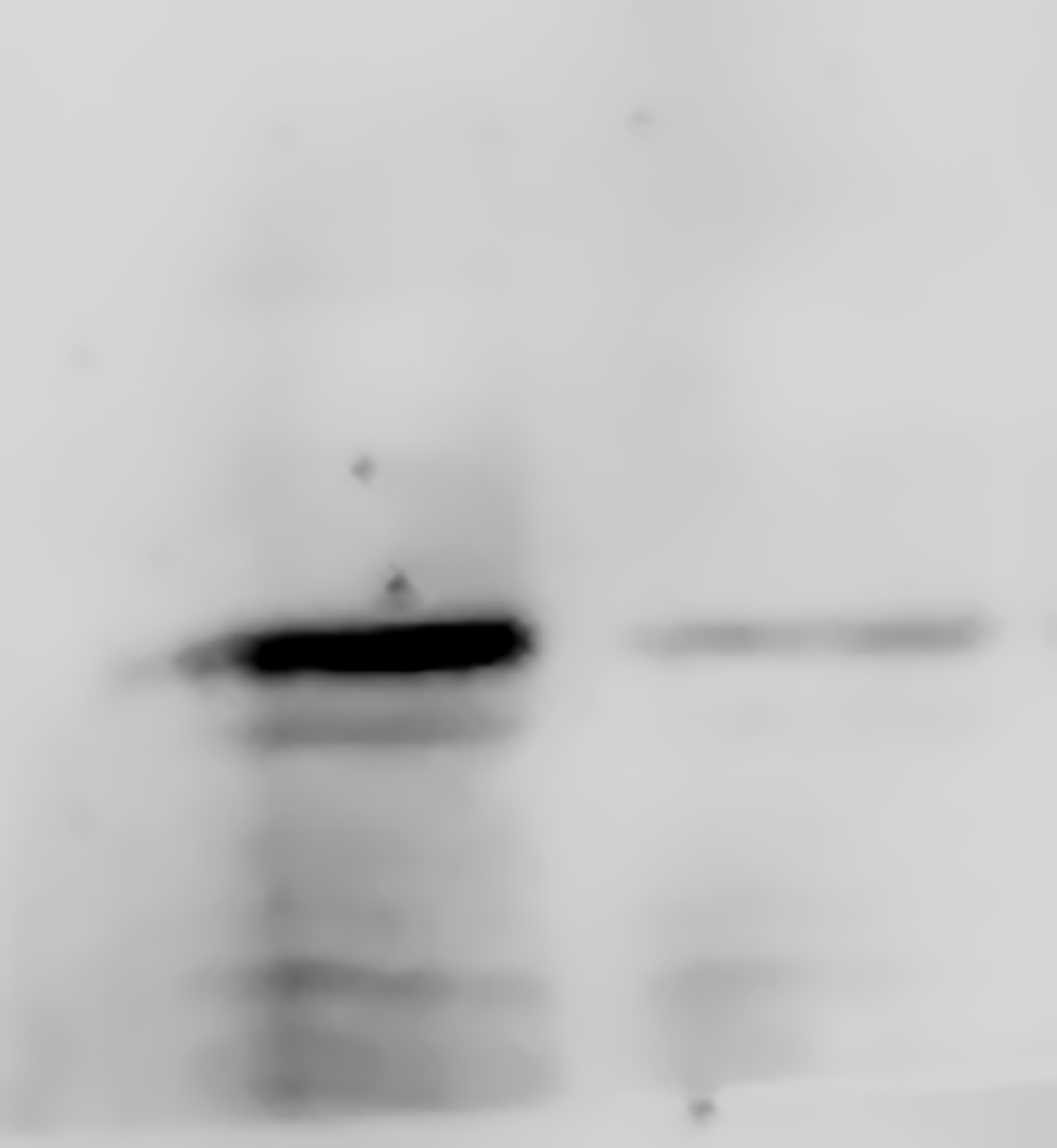

Supplement: Supplementary file 2 [file Data_Sheet_1.ZIP › Western Blot/Film/SIRT1.tif]

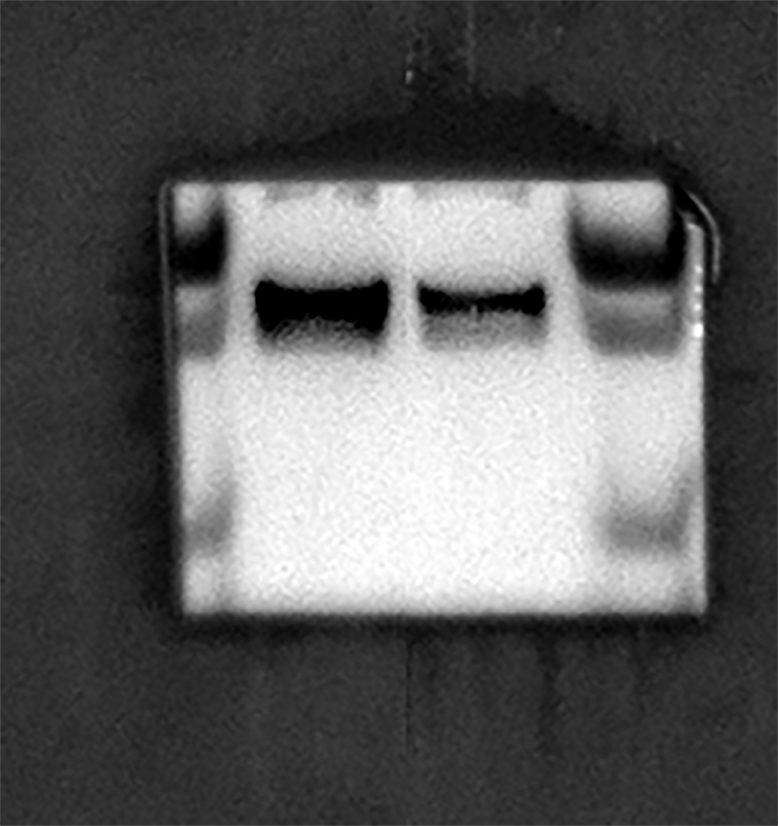

Supplement: Supplementary file 2 [file Data_Sheet_1.ZIP › Western Blot/Film/Smad3.tif]

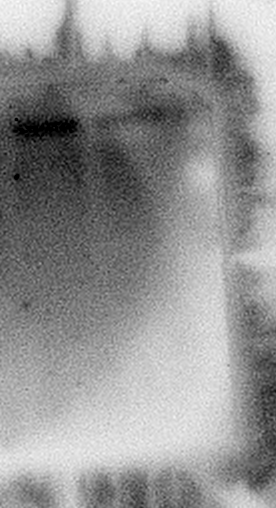

Supplement: Supplementary file 2 [file Data_Sheet_1.ZIP › Western Blot/Film/TGF-a┬1.tif]

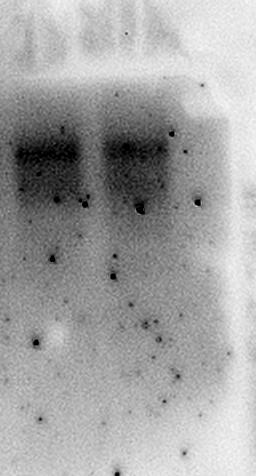

Supplement: Supplementary file 2 [file Data_Sheet_1.ZIP › Western Blot/Film/TNF-a┴.tif]

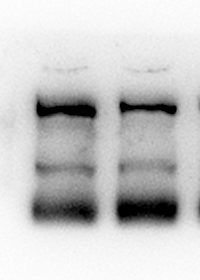

Supplement: Supplementary file 2 [file Data_Sheet_1.ZIP › Western Blot/Film/ZO-1.tif]

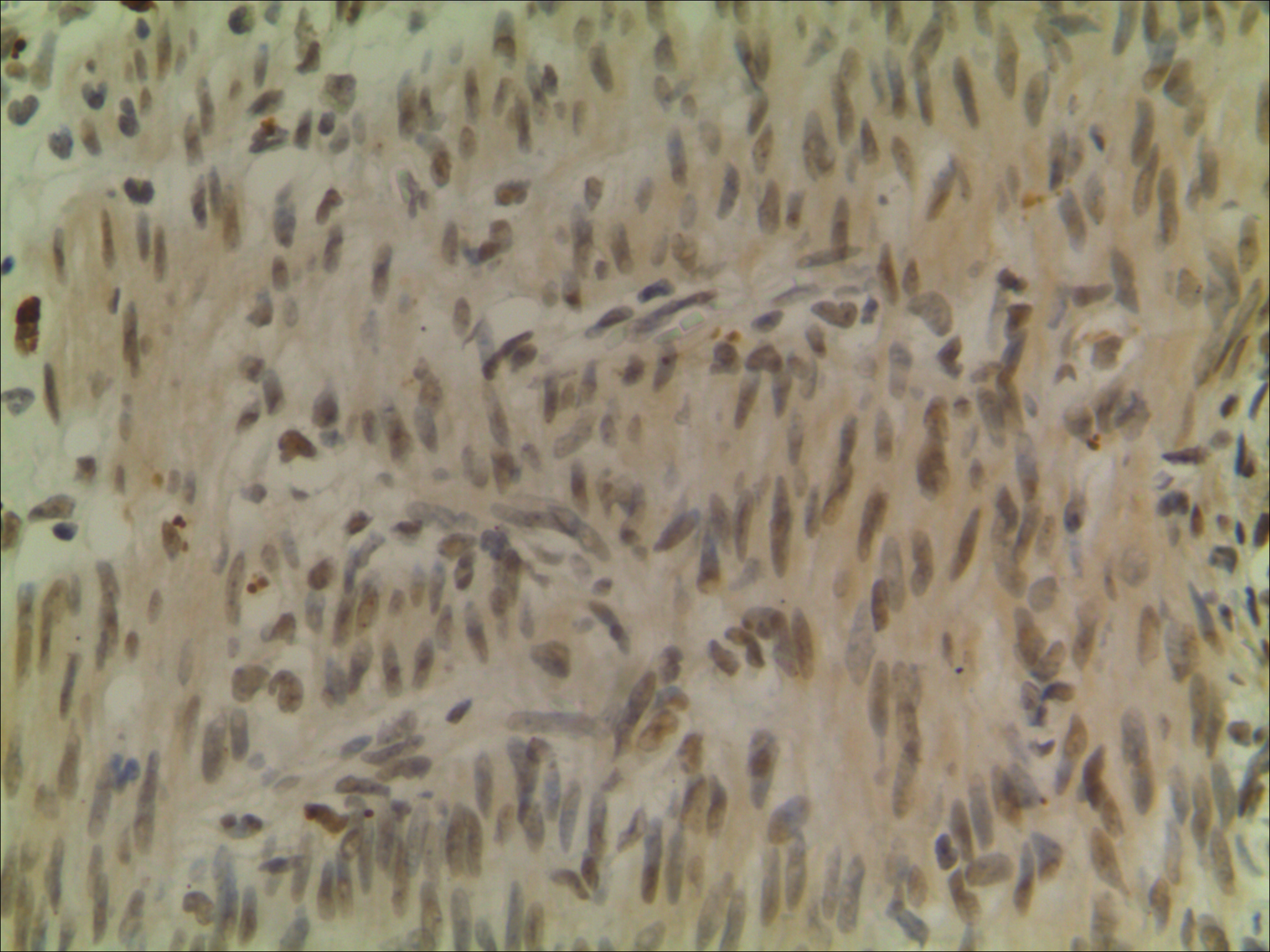

Supplement: Supplementary file 4 [file Data_Sheet_3.ZIP › Enterostenosis SIRT1 in muscular layer.tif]

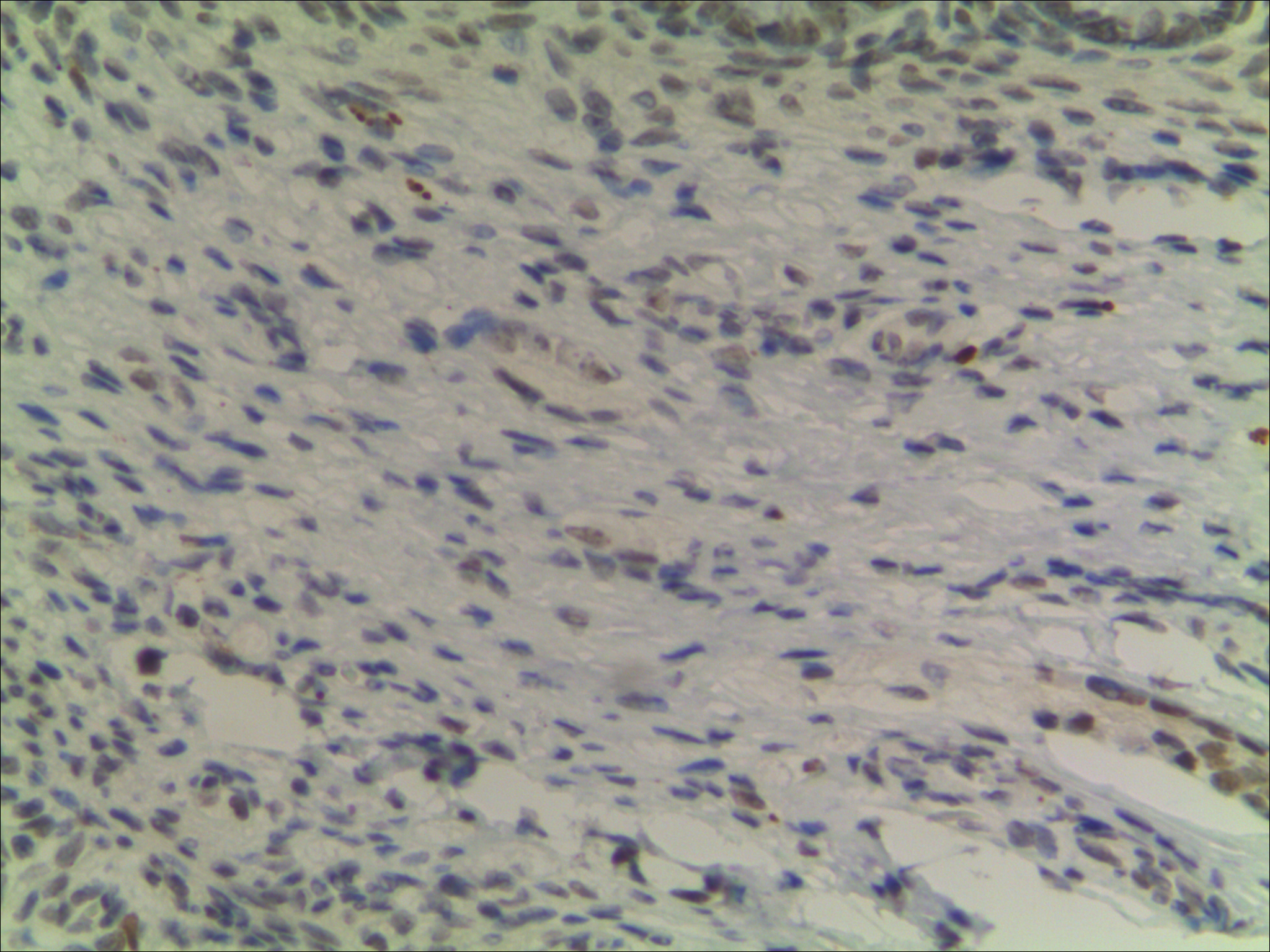

Supplement: Supplementary file 4 [file Data_Sheet_3.ZIP › Enterostenosis SIRT1 in submucosa.tif]

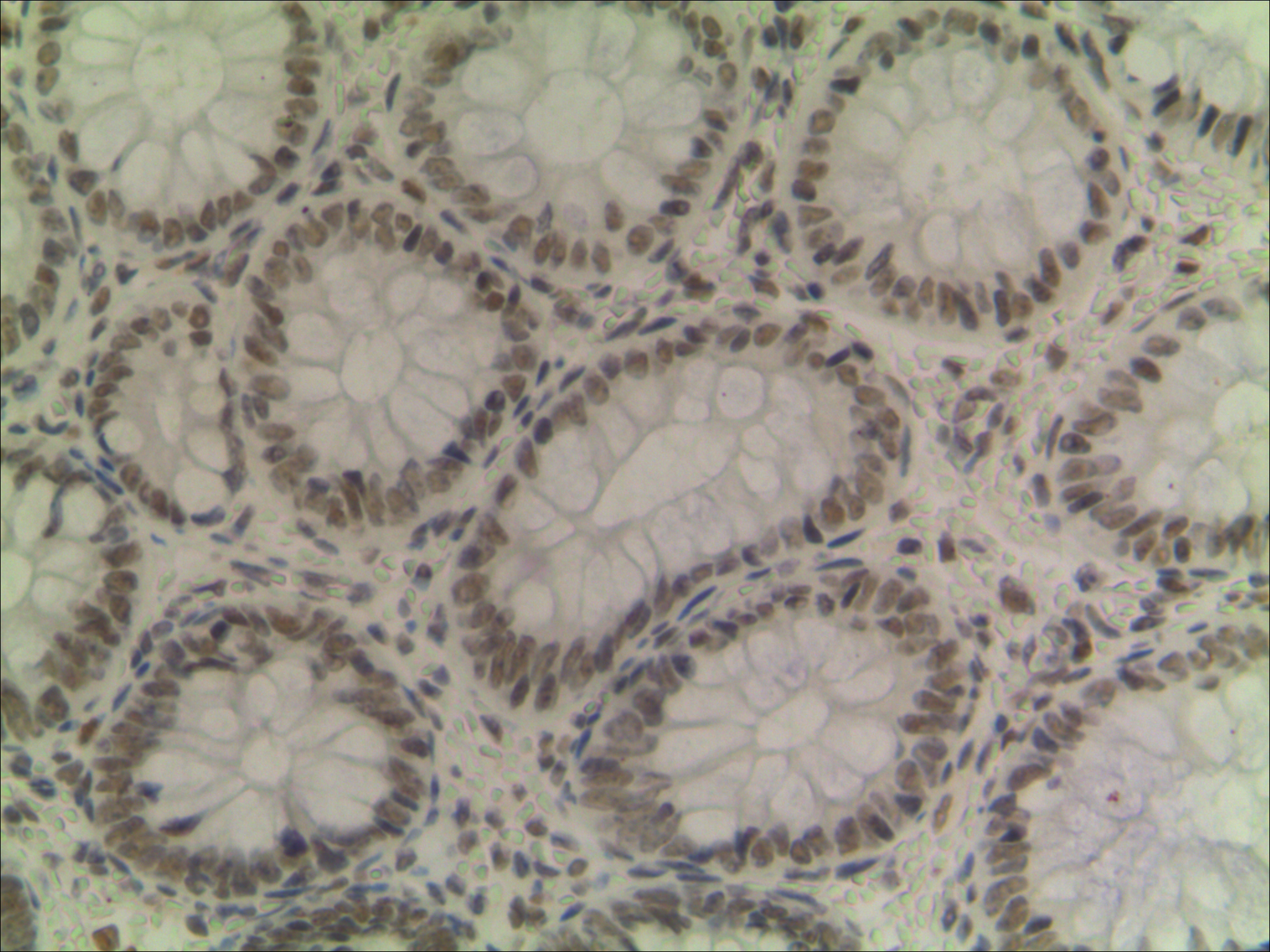

Supplement: Supplementary file 4 [file Data_Sheet_3.ZIP › Enterostenosis SIRT1 in mucous layer.tif]

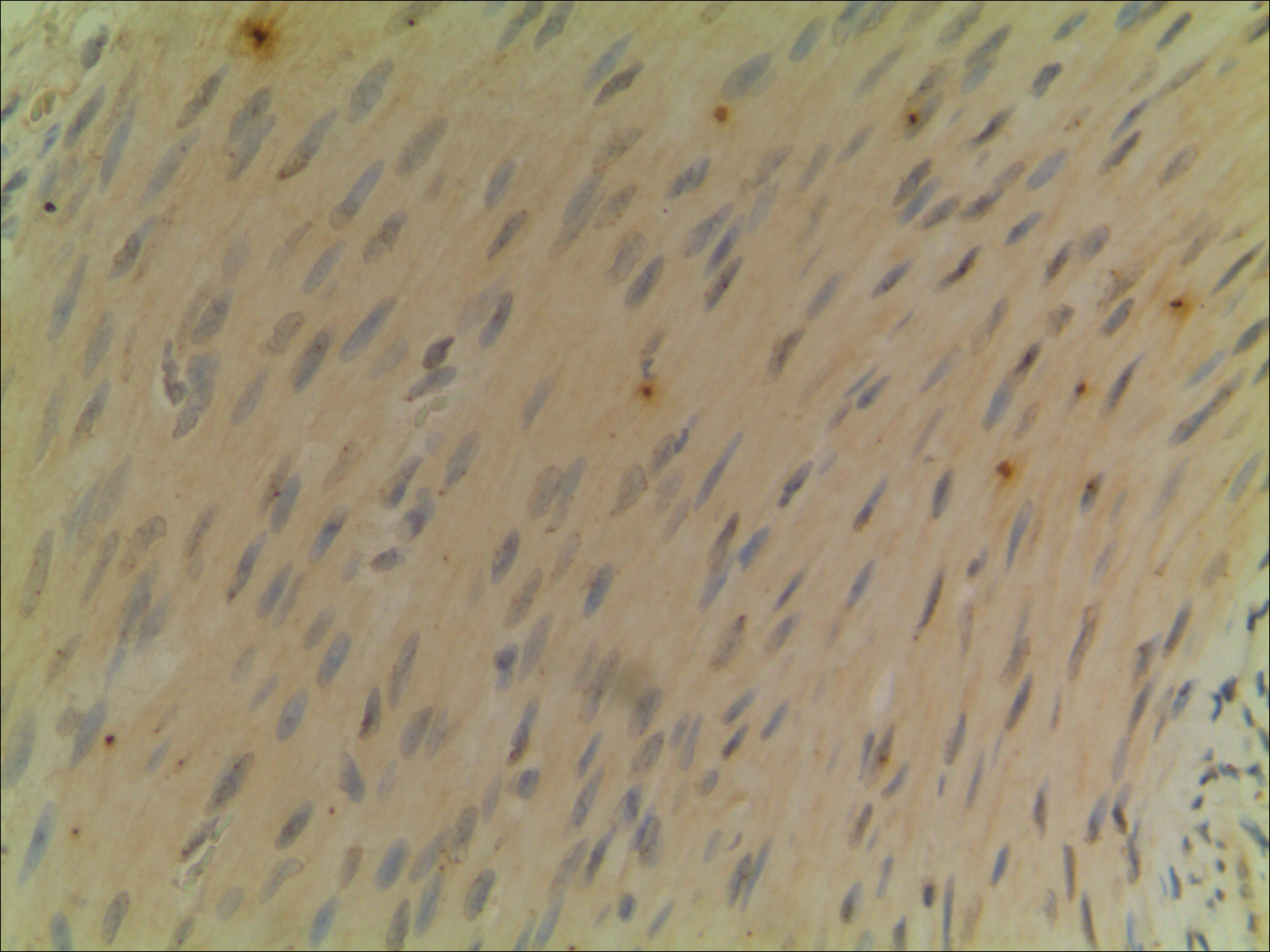

Supplement: Supplementary file 5 [file Data_Sheet_4.ZIP › Enterostenosis TGF-a┬1 in muscular layer.tif]

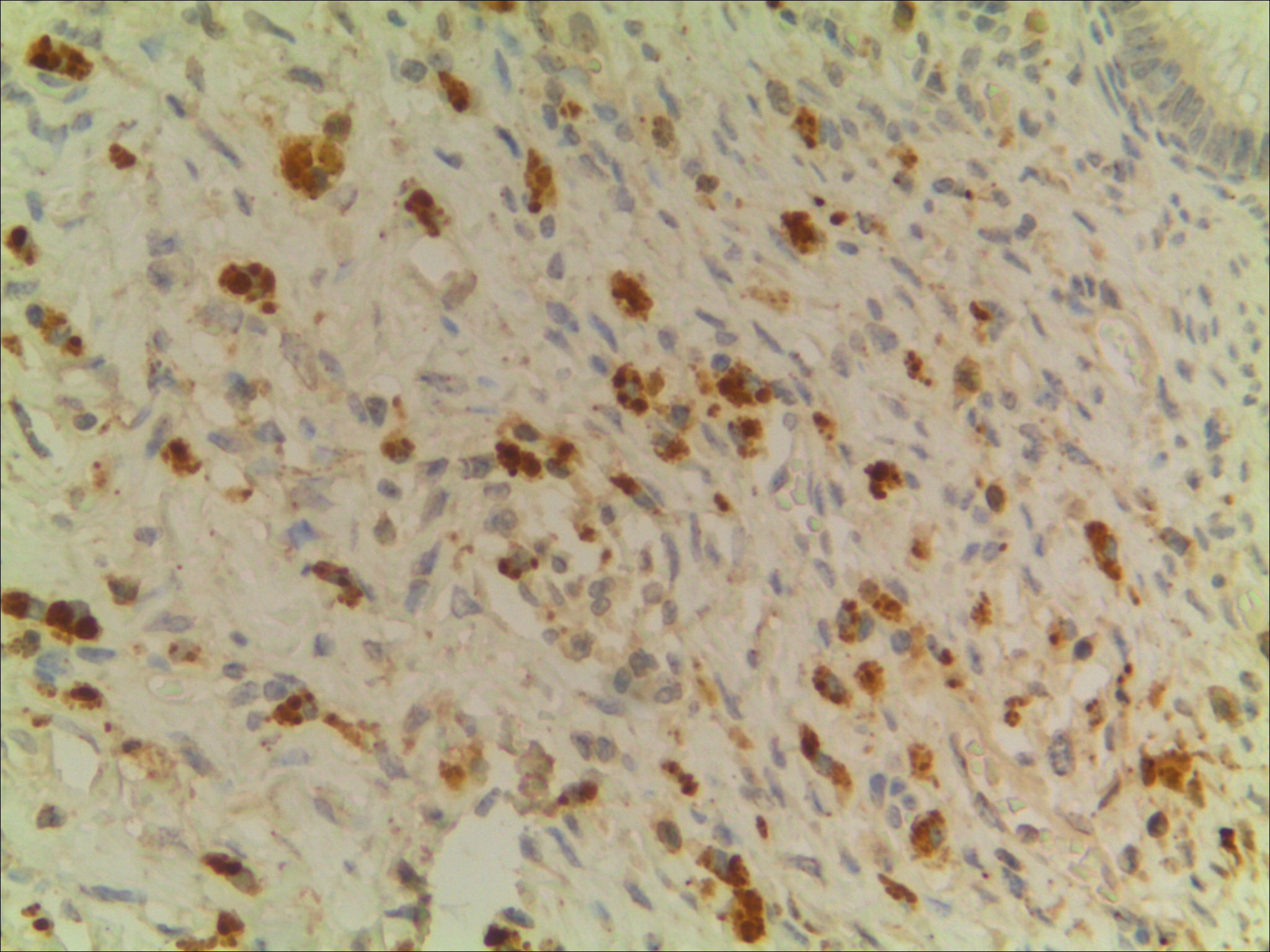

Supplement: Supplementary file 5 [file Data_Sheet_4.ZIP › Enterostenosis TGF-a┬1 in submucosa.tif]

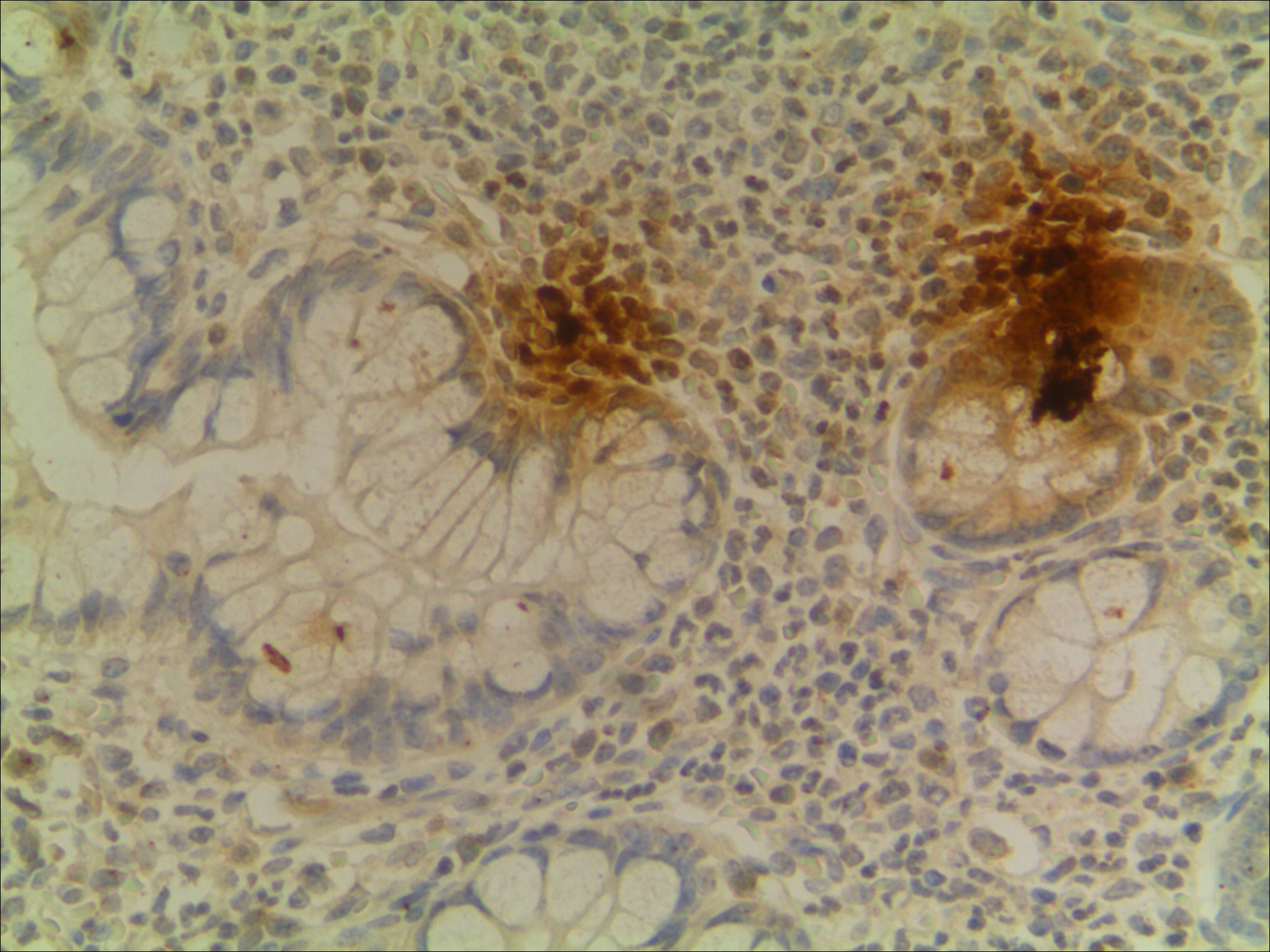

Supplement: Supplementary file 5 [file Data_Sheet_4.ZIP › Enterostenosis TGF-a┬1 in mucous layer.tif]

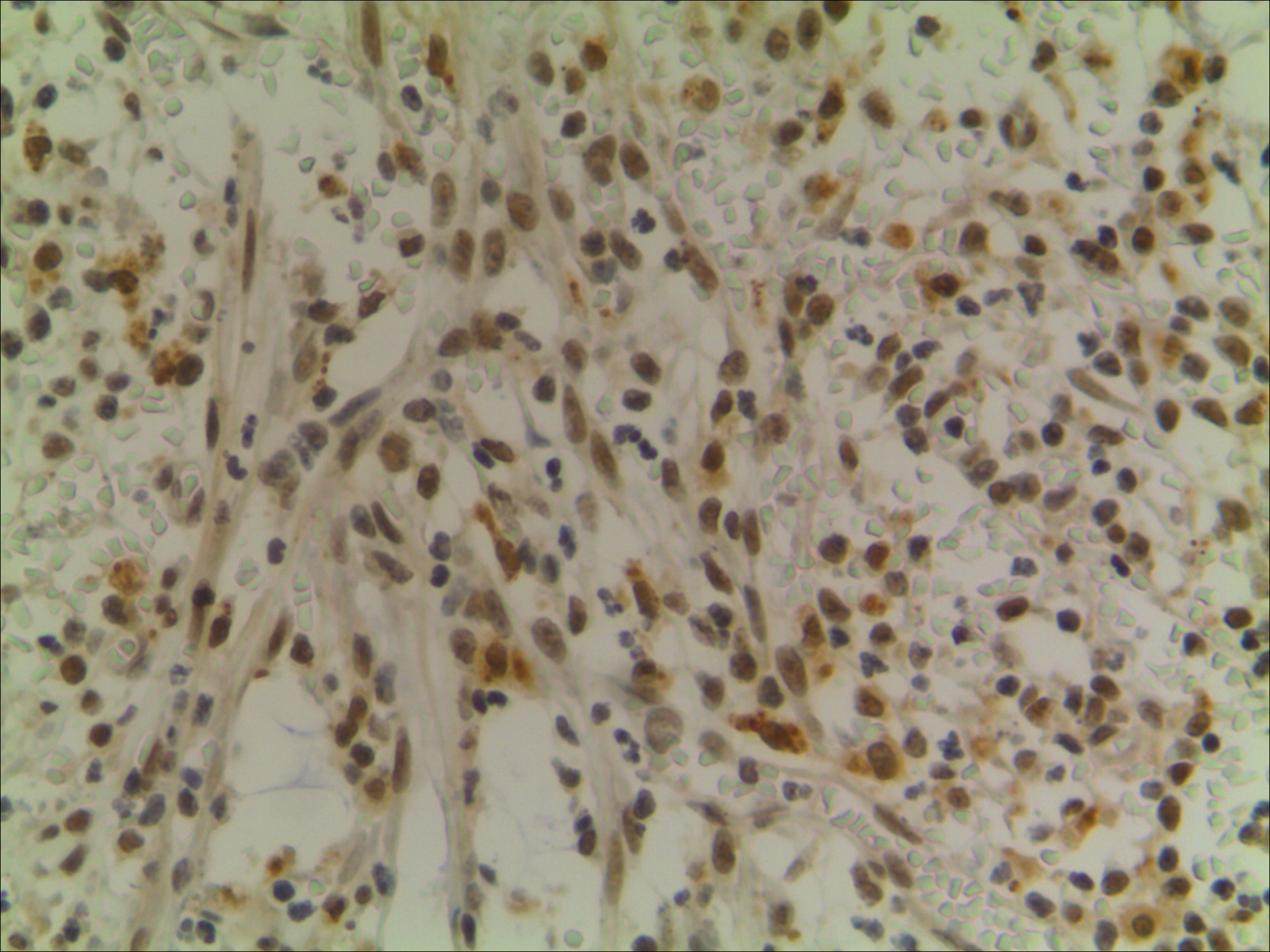

Supplement: Supplementary file 6 [file Data_Sheet_5.ZIP › Normal SIRT1 in submucosa.tif]

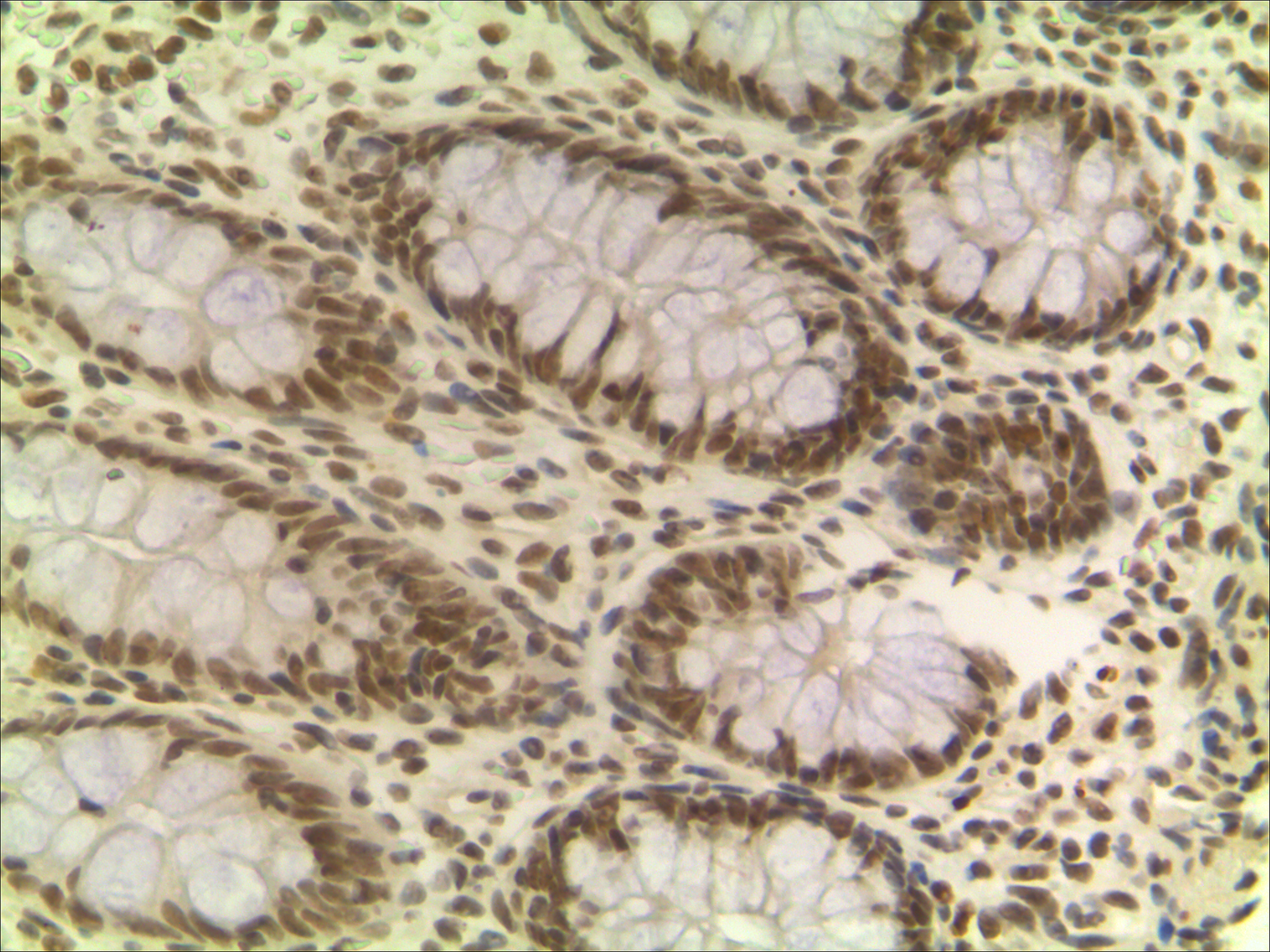

Supplement: Supplementary file 6 [file Data_Sheet_5.ZIP › Normal SIRT1 mucous layer.tif]

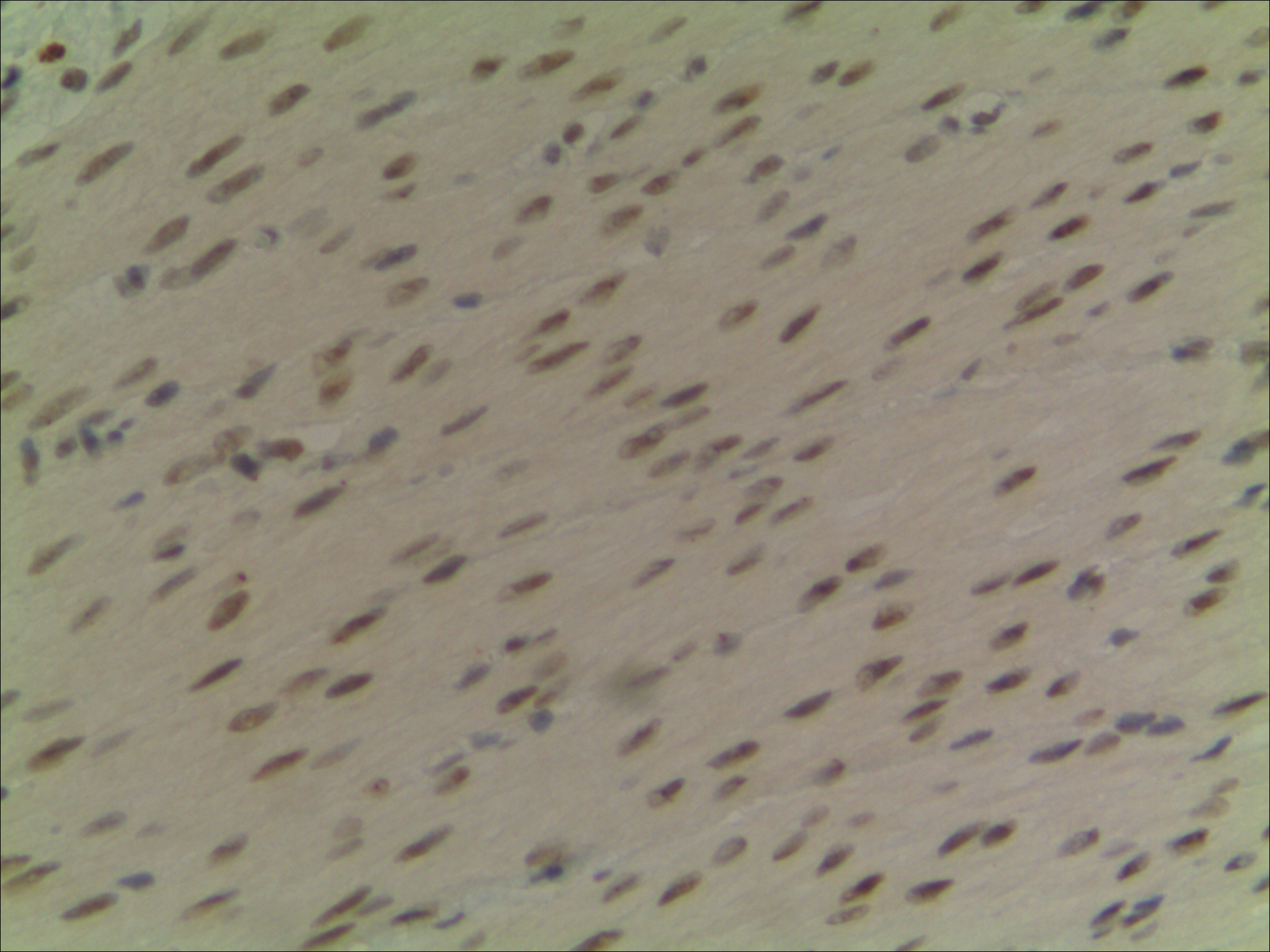

Supplement: Supplementary file 6 [file Data_Sheet_5.ZIP › Normal SIRT1 in muscular layer.tif]

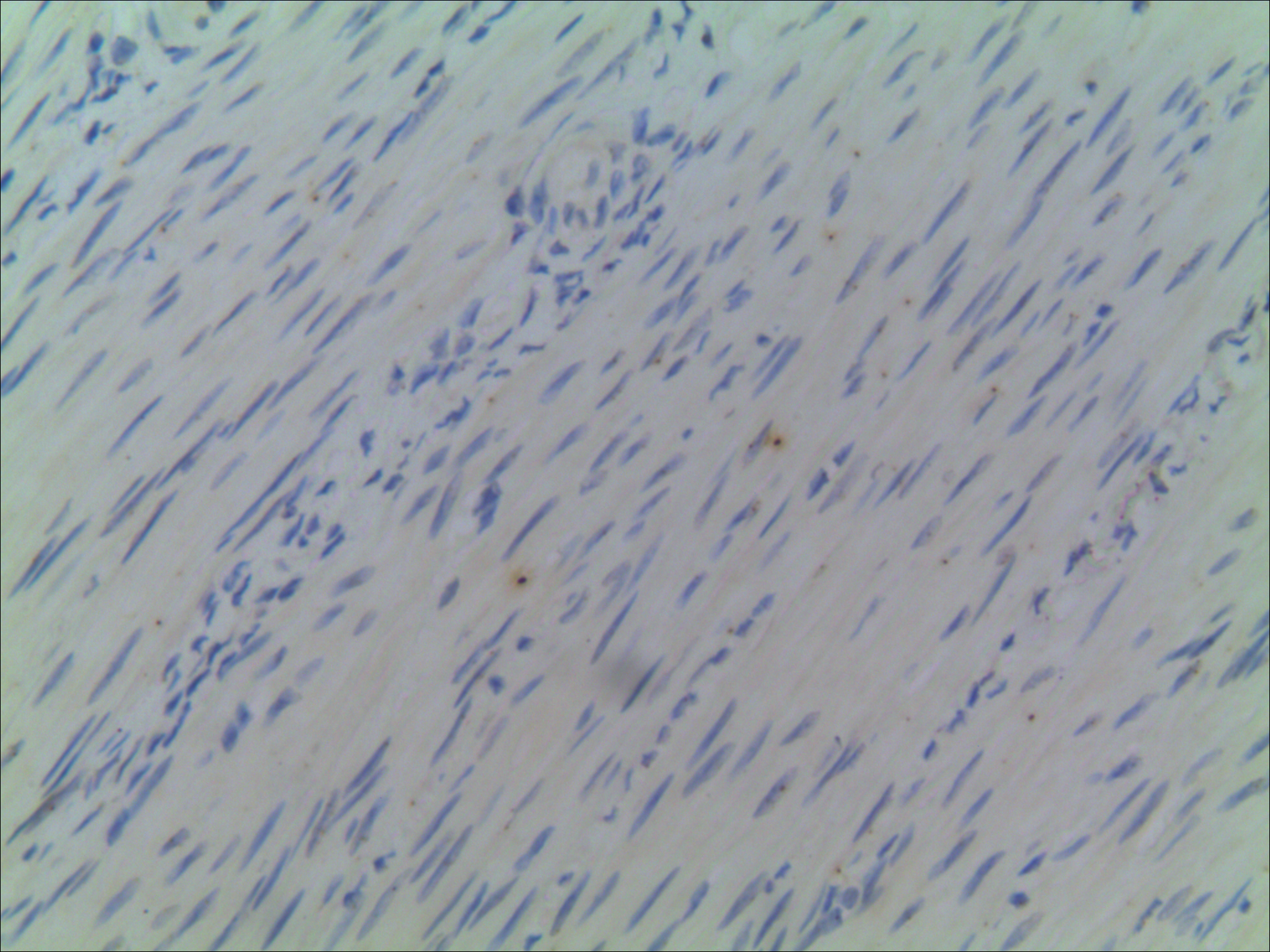

Supplement: Supplementary file 7 [file Data_Sheet_6.ZIP › Normal TGF-a┬1 in muscular layer.tif]

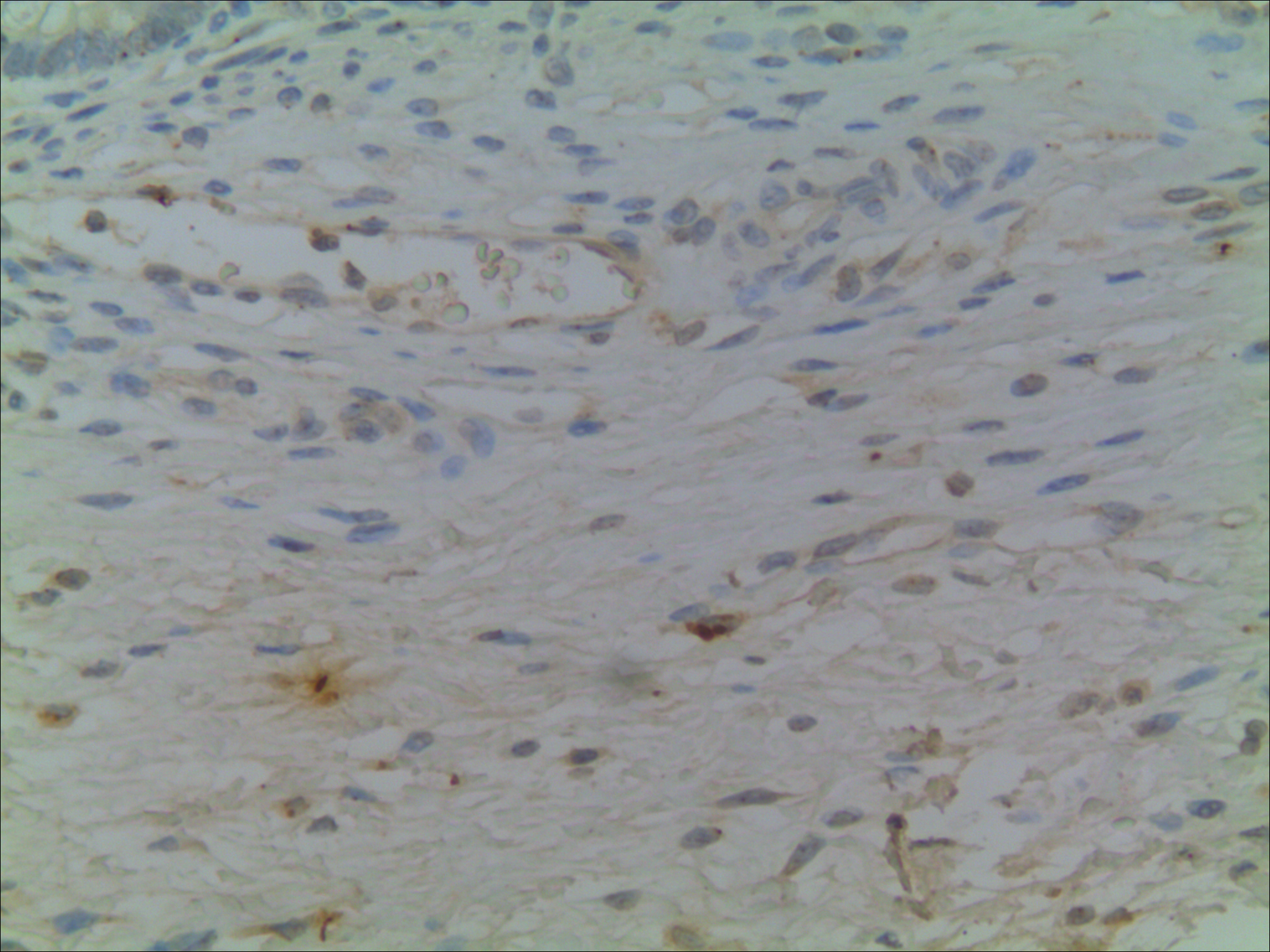

Supplement: Supplementary file 7 [file Data_Sheet_6.ZIP › Normal TGF-a┬1 in submucosa.tif]

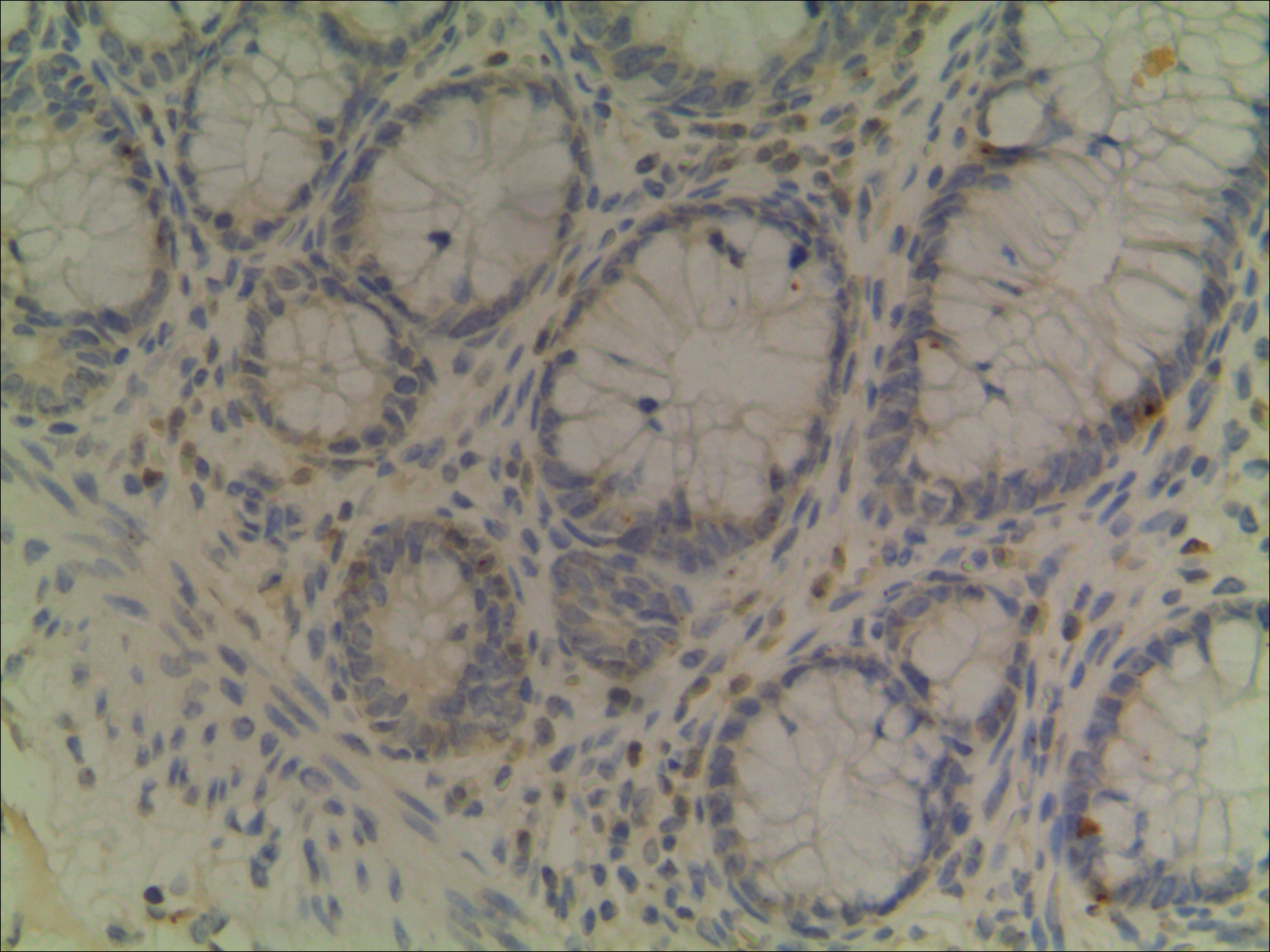

Supplement: Supplementary file 7 [file Data_Sheet_6.ZIP › Normal TGF-a┬1 in mucous layer.tif]
